# Gold(I)-Triphenylphosphine Complexes with Hypoxanthine-Derived Ligands: *In Vitro* Evaluations of Anticancer and Anti-Inflammatory Activities

**DOI:** 10.1371/journal.pone.0107373

**Published:** 2014-09-16

**Authors:** Radka Křikavová, Jan Hošek, Ján Vančo, Jakub Hutyra, Zdeněk Dvořák, Zdeněk Trávníček

**Affiliations:** 1 Regional Centre of Advanced Technologies and Materials & Department of Inorganic Chemistry, Faculty of Science, Palacký University, Olomouc, Czech Republic; 2 Regional Centre of Advanced Technologies and Materials & Department of Cell Biology and Genetics, Faculty of Science, Palacký University, Olomouc, Czech Republic; Virginia Commonwealth University, United States of America

## Abstract

A series of gold(I) complexes involving triphenylphosphine (PPh_3_) and one *N*-donor ligand derived from deprotonated mono- or disubstituted hypoxanthine (HL_n_) of the general composition [Au(L_n_)(PPh_3_)] (**1**–**9**) is reported. The complexes were thoroughly characterized, including multinuclear high resolution NMR spectroscopy as well as single crystal X-ray analysis (for complexes **1** and **3**). The complexes were screened for their *in* *vitro* cytotoxicity against human cancer cell lines MCF7 (breast carcinoma), HOS (osteosarcoma) and THP-1 (monocytic leukaemia), which identified the complexes **4**–**6** as the most promising representatives, who antiproliferative activity was further tested against A549 (lung adenocarcinoma), G-361 (melanoma), HeLa (cervical cancer), A2780 (ovarian carcinoma), A2780R (ovarian carcinoma resistant to *cisplatin*), 22Rv1 (prostate cancer) cell lines. Complexes **4**–**6** showed a significantly higher *in* *vitro* anticancer effect against the employed cancer cells, except for G-361, as compared with the commercially used anticancer drug *cisplatin*, with IC_50_ ≈ 1–30 µM. Anti-inflammatory activity was evaluated *in* *vitro* by the assessment of the ability of the complexes to modulate secretion of the pro-inflammatory cytokines, i.e. tumour necrosis factor-α (TNF-α) and interleukin-1β (IL-1β), in the lipopolysaccharide-activated macrophage-like THP-1 cell model. The results of this study identified the complexes as auspicious anti-inflammatory agents with similar or better activity as compared with the clinically applied gold-based antiarthritic drug Auranofin. In an effort to explore the possible mechanisms responsible for the biological effect, the products of interactions of selected complexes with sulfur-containing biomolecules (L-cysteine and reduced glutathione) were studied by means of the mass-spectrometry study.

## Introduction

**Figure 1:**
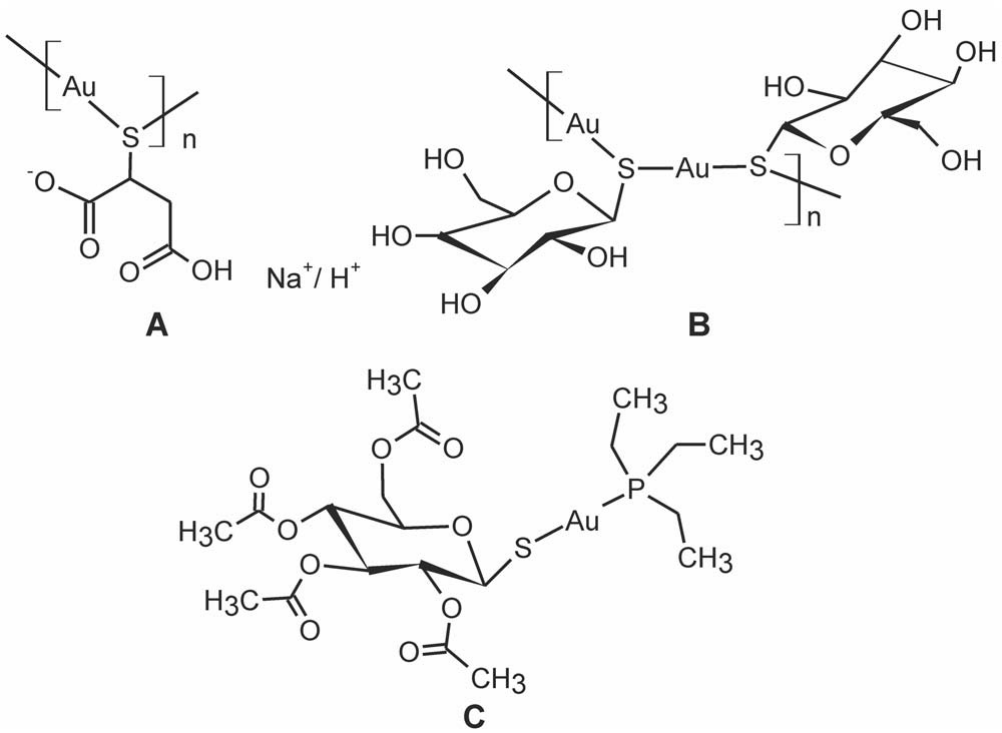
Schematic representations of gold(I) complexes used as anti-inflammatory drugs. **A**: Sodium aurothiomalate (Myochrysin, sodium {(2-carboxy-1-carboxylatoethyl)thiolato}gold(I)); **B**: Aurothioglucose (Solganol, {(2 *S*,3*R*,4 *S*,5 *S*,6*R*)-3,4,5-trihydroxy-6-(hydroxymethyl)-oxane-2-thiolato}gold(I)); **C**: Auranofin (Ridaura, triethylphosphine-(2,3,4,6-tetra-*O*-acetyl-1-D-thiopyranosato-*S*)gold(I)).

Gold-based medication was used for a wide range of ailments already in the distant history of ancient China 2500 BC [1], [2]. Thus, it is quite extraordinary that even current clinical practice still recognizes chrysotherapy (*chrysos*, gold in Greek), i.e. the treatment of diseases by the administration of gold-based therapeutic agents, as an integral part of modern medicine [3], [4]. During the last eighty years of clinical use of gold-based metallotherapeutics, two major groups of complexes have been introduced. The first one is represented by the parenteral preparations, containing relatively simple gold(I)-complexes, such as sodium aurothiomalate (Myochrysin, sodium ((2-carboxy-1-carboxylatoethyl)thiolato)gold(I)) and aurothioglucose (Solganol, {(2 *S*,3*R*,4 *S*,5 *S*,6*R*)-3,4,5-trihydroxy-6-(hydroxymethyl)-oxane-2-thiolato}gold(I)) [5], and the second one comprises the orally administered drug Auranofin (Ridaura) [6]–[8] (triethylphosphine-(2,3,4,6-tetra-*O*-acetyl-1-D-thiopyranosato-*S*)gold(I)) (See Figure 1). Nowadays, the gold-based metallotherapeutics do not represent antiarthritic agents of the first choice, since new highly potent and more specific anti-rheumatic compounds have been developed (i.e. biopharmaceuticals, novel non-steroidal anti-inflammatory drugs, NSAIDs, or corticosteroids), however, they are still indicated in specific conditions, including moderately to severely active rheumatoid arthritis [8], [9]. Although the clinical application of Auranofin has declined in recent years, there are several attempts for repurposing it in other significant indications, such as cytoprotective agents and in the treatment of HIV, severe microbial infections and cancer [1], [2], [4], [8], [10].

Several Auranofin inspired gold(I)-complexes have been studied for significant anti-inflammatory, and/or antitumor activities. Among linear gold(I) phosphine complexes, those involving ligands as e.g. dithiocarbamates [1], sulfanylpropenoates [11], naphthalimide derivatives [12], imidazole, pyrazole [13], [14] as well as purine derivatives [15], [16] have been investigated. It has been established that the gold(I)-phosphine moiety is responsible for the actual interactions [3], [17] with the target sites of the biological molecules, i.e. mostly the selanyl-groups of enzymes [1], [18], while the other, weaker bonded ligand influences the kinetic profile of the compounds. This principle has been employed by our group in several recently published works, describing highly antitumor active as well as anti-inflammatory potent gold(I) complexes of the type [Au(L)(PPh_3_)] involving various *N*6-benzyladenine derivatives (L) [15], [16].

**Figure 2:**
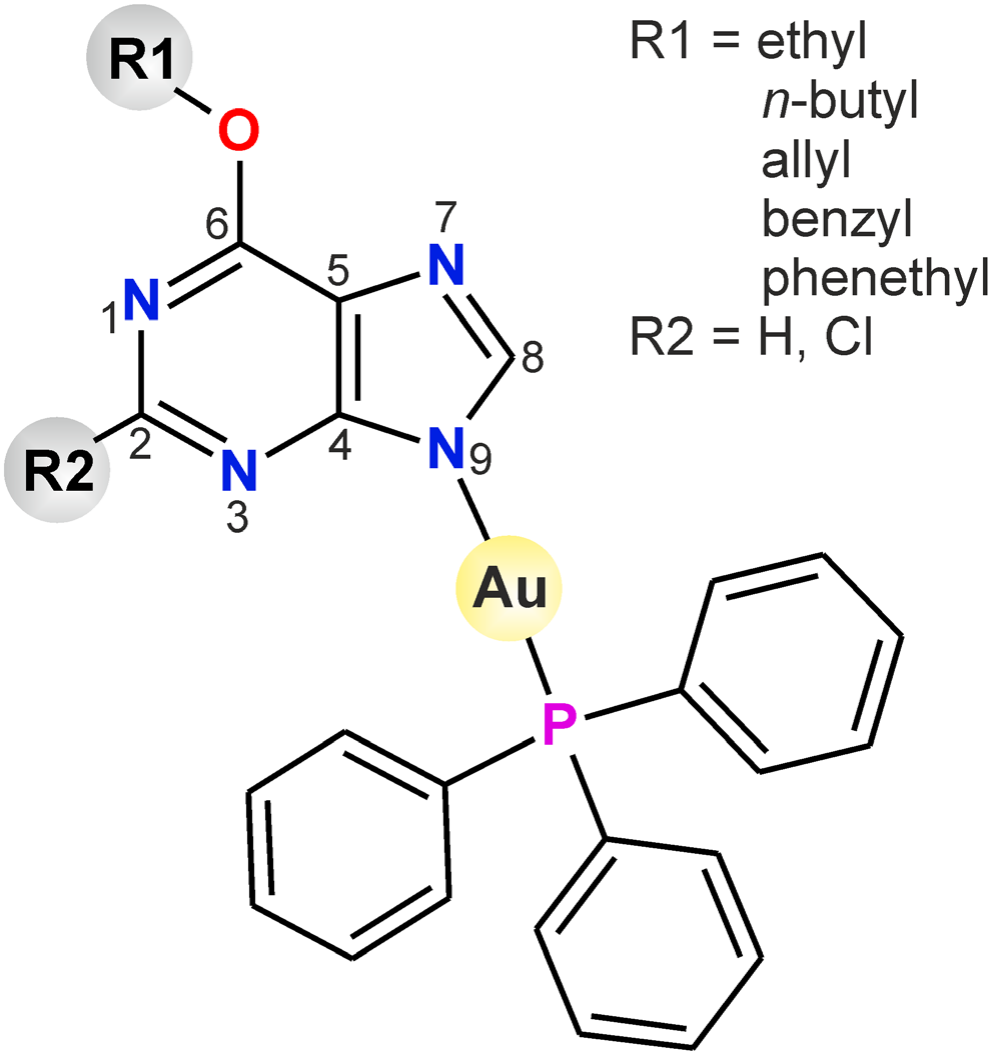
Schematic representation of the prepared gold(I) complexes 1–9.

In this work, we report a series of gold(I) complexes of the general formula [Au(L_n_)(PPh_3_)], where HL_n_ represents variously mono- and disubstituted derivatives of hypoxanthine (See Figure 2). The rationale for the selection of these *N*-donor ligands is based on the fact that variously substituted derivatives of hypoxanthine have been identified as promising inhibitors of diverse essential enzymes, as cyclin-dependent kinases [19], [20] and *O*6-alkylguanine-DNA-alkyltransferase [21], [22], and therefore in combination with the {Au-PPh_3_} moiety could bring additional means of influencing the cellular metabolism both on the level of cell division, and modulation of cellular responses to the inflammatory stimuli.

The complexes were fully characterized, including high resolution NMR spectroscopy and single crystal X-ray analysis (for complexes **1** and **3**), and were evaluated for their cytotoxicity against a panel of nine human cancer cell lines (MCF7, HOS, THP-1, A549, G361, HeLa, A2780, A2780R (*cisplatin* resistant), and 22Rv1, and also for their *in* *vitro* anti-inflammatory effect on the model of LPS-stimulated human monocytic leukaemia (THP-1) macrophage-like cell line and the mechanisms of interaction of the complexes with sulfur-containing biomolecules (i.e. L-cysteine and reduced glutathione) were studied by means of mass spectrometry. Moreover, this work fits within the focus of our long term study dedicated to research and development of new transition metal complexes showing various types of biological effects, represented dominantly by the anticancer {Pt(II) [23], Pd(II) [24], Cu(II) [25], [26] or Ru(III) [27]}, anti-inflammatory {Au(I/III)} [15], [16], [28], antidiabetic and cytoprotective {Cu(II) [29]} activities.

## Materials and Methods

### Chemicals and Biochemicals

All the chemicals, involving H[AuCl_4_]·3H_2_O (Acros Organics, Pardubice, Czech Republic), triphenylphosphine (PPh_3_; Sigma-Aldrich Co., Prague, Czech Republic), NaOH (Sigma-Aldrich Co., Prague, Czech Republic) and used solvents (acetone, diethyl ether, dimethyl sulfoxide (DMSO), toluene, *n*-hexane and *N, N^’^*-dimethylformamide (DMF); Fisher-Scientific, Pardubice, Czech Republic) were obtained from the commercial sources and were used without any further purification. The *N*-donor ligands derived from hypoxanthine, HL_1–9_ (HL_1_ = 6-ethoxy-9*H*-purine; HL_2_ = 6-butyloxy-9*H*-purine; HL_3_ = 6-allyloxy-9*H*-purine; HL_4_ = 6-benzyloxy-9*H*-purine; HL_5_ = 6-phenetyloxy-9*H*-purine; HL_6_ = 2-chloro-6-ethoxy-9*H*-purine; HL_7_ = 2-chloro-6-butyloxy-9*H*-purine, HL_8_ = 2-chloro-6-allyloxy-9*H*-purine, HL_8_ = 2-chloro-6-benzyloxy-9*H*-purine) were prepared according to the previously published procedure [19] and characterized by elemental analysis, FT-IR and ^1^H and ^13^C NMR spectroscopy, which gave evidence about their composition and purity. The starting [AuCl(PPh_3_)] complex was synthesized as described previously [30], [31].

The RPMI 1640 medium and penicillin-streptomycin mixture (Lonza, Verviers, Belgium), phosphate-buffered saline (PBS), foetal bovine serum (FBS), phorbol myristate acetate (PMA), prednisone (≥98%), Auranofin (≥98%), erythrosin B, and *Escherichia coli* 0111:B4 lipopolysaccharide (LPS) (Sigma-Aldrich, Steinheim, Germany), as well as a Cell Proliferation Reagent WST-1, and cOmplete Proteinase Inhibitor Cocktail (Roche, Mannheim, Germany) were obtained from commercial sources.

A RealTime Ready Cell Lysis Kit (Roche, Mannheim, Germany) served for isolation of RNA from cells and Transcriptor Universal cDNA Master (Roche, Mannheim, Germany) was used for reverse transcription of RNA to cDNA. Specific primers and probes (Gene Expression assays) for polymerase chain reaction (PCR) were obtained from Applied Biosystems (Foster City, CA, USA). The following assays were chosen for the quantification of gene expression: Hs00174128_m1 for tumour necrosis factor-α (TNF-α), Hs01555410_m1 for interleukin-1β (IL-1β), and 4326315E for β-actin, which served as an internal control of gene expression. Quantitative PCR (qPCR) was performed with Fast Start Universal Probe Master (Roche, Mannheim, Germany). Instant ELISA Kits (eBioscience, Vienna, Austria) were used to evaluate the production of TNF-α and IL-1β by the enzyme linked immunosorbent assay (ELISA). The Immun-Blot PVDF (polyvinylidene fluoride) membrane 0.2 µm (Bio-Rad, Hercules, CA, USA) and albumin bovine fraction V (BSA) (Serva, Heidelberg, Germany) were used for Westernblot. Murine monoclonal anti-I*κ*B-α (Cell Signaling, Danvers, MA, USA), murine monoclonal anti-β-actin (Abcam, Cambridge, UK) and goat polyclonal anti-mouse IgG (with the conjugated peroxidase) antibodies (Sigma-Aldrich, Saint Louis, MO, USA) were applied for immunodetection. The activity of the conjugated peroxidase was detected by an Opti-4CN Substrate Kit (Bio-Rad, Hercules, CA, USA).

### Chemistry

Gold(I) complexes of the general formula [Au(L_n_)(PPh_3_)] (**1**–**9**), where L_n_ stands for a deprotonated form of a hypoxanthine-derived compound, were prepared by a slight modification of the previously reported procedure for the synthesis of similar [Au(L)(PPh_3_)] complexes, in which HL stands for 6-benzylaminopurine derivatives [15], [16]. Concretely, the reactions of the acetone solutions of [AuCl(PPh_3_)] (1 mmol in 10 mL) were mixed with the corresponding hypoxanthine derivative (HL_n_; 1 mmol in 20 mL), and subsequently, 1 mL of 1 M NaOH was added into the reaction mixture. The mixture was stirred for 3 h, after which precipitated NaCl was filtered off. The colourless filtrate was evaporated to dryness and the residue was collected. Recrystallization was carried out from acetone. In the case when the residue was of gel-like consistency, 20 mL of diethyl ether was added and the suspension was sonicated to obtain a powder product. Crystals of complexes **1** and **3**, suitable for single crystal X-ray analysis, were prepared by a slow diffusion of *n*-hexane into a saturated solution of the appropriate complex in toluene. The purity and composition of the obtained gold(I) complexes **1**–**9** were established based on the methods of elemental analysis, electrospray-ionization mass spectrometry (ESI-MS), ^1^H and ^13^C NMR, and FT-IR spectroscopies, and thermal analysis (TG/DTA) (characterization data are given in File S1). The molecular and crystal structures were determined by single crystal X-ray analysis for complexes **1** and **3**.

### Physical Measurements

Elemental analyses (C, H, N) were carried out on a Flash 2000 CHNO-S Analyser (Thermo Scientific, USA). FT-IR spectra were measured on a Nexus 670 FT-IR spectrometer (ThermoNicolet, USA) by the ATR technique in the 200–4000 cm^−1^ range. ^1 ^H, ^13^C and ^31^P NMR spectra were measured in DMF-*d*_7_ on a Varian 400 MHz NMR spectrometer at 300 K using tetramethylsilane (SiMe_4_) (for ^1^H and ^13^C spectra) and 85% H_3_PO_4_ (for ^31^P) as an internal reference standard. Mass spectra of methanol solutions of complexes were recorded on an LCQ Fleet Ion-Trap mass spectrometer using the positive mode electrospray ionization (Thermo Scientific, USA). Thermogravimetric (TG) and differential thermal (DTA) analyses were performed on a thermal analyser Exstar TG/DTA 6200 (Seiko Instruments Inc., Japan) in dynamic air conditions (50 mL min^−1^) between room temperature (ca 25°C) and 950°C (gradient 2.5°C min^−1^). Single crystal X-ray analyses were performed on an Xcalibur2 diffractometer equipped with a CCD detector Sapphire2 (Oxford Diffraction Ltd., Oxford, UK) using MoKα radiation (monochromator Enhance, Oxford Diffraction Ltd.), and using the ω-scan technique at 120 K. Data collection, data reduction and cell parameter refinements were performed by the *CrysAlis* software package [32]. The molecular structures were solved by direct methods and all non-hydrogen atoms were refined anisotropically on *F^2^* using the full-matrix least-squares procedure (*SHELX-97*) [33]. H-atoms were found in difference maps and refined by using the riding model with C−H = 0.95, 0.98 and 0.99 Å, with *U*_iso_(H) = 1.2*U*_eq_(CH, CH_2_) and 1.5*U*_eq_(CH_3_). The highest residual peaks of 2.623 e Å^–3^ and 1.792 e Å^–3^ were located 0.85 Å from Au1 (for complex **1**), and 0.88 Å from Au1 (for complex **3**), respectively. Molecular graphics were drawn and additional structural parameters were interpreted in *DIAMOND* [34] and *Mercury* [35].

### Maintenance and Preparation of Macrophages

The human monocytic leukaemia cell line THP-1 (ECACC, Salisbury, UK) was used for the *in* *vitro* anti-inflammatory activity evaluation. The cells were cultivated at 37°C in the RPMI 1640 medium supplemented with 2 mM l-glutamine, 10% FBS, 100 U/mL of penicillin and 100 µg/mL of streptomycin in humidified atmosphere containing 5% CO_2_. Stabilized cells (3^rd^–15^th^ passage) were split into microtitration plates to get the concentration of 500 000 cells/mL and the differentiation to macrophages was induced by addition of phorbol myristate acetate (PMA) dissolved in DMSO at the final concentration of 50 ng/ml and the cells were incubated for 24 h. Unlike monocytes, differentiated macrophages tend to adhere to the bottom of the cultivation plates. For the subsequent 24 h, the cells were incubated with the fresh complete RPMI medium, i.e. containing antibiotics and FBS, without PMA. Then, the medium was aspirated, and the cells were washed with PBS and cultivated in the serum-free RPMI 1640 medium for next 24 h. These prepared macrophages were used for the detection of inflammatory response.

### *In Vitro* Cytotoxicity Testing

Cytotoxicity in THP-1 cells was determined by the WST-1 assay. The THP-1 cells (floating monocytes, 500 000 cells/mL) were incubated in 100 µL of the serum-free RPMI 1640 medium and seeded into 96-well plates in triplicate at 37°C. Measurements were taken 24 h after the treatment with the tested compounds dissolved in 0.1% DMSO in the concentration range of 0.16–10 µM. Viability was determined by the WST-1 test according to the manufacturer’s manual. The amount of created formazan (correlating with the number of metabolically active cells in the culture) was calculated as a percentage of the control cells, which were treated only with 0.1% DMSO and was set-up as 100%. The cytotoxic IC_50_ values of the tested compounds were calculated from the obtained data. The WST-1 assay was performed spectrophotometrically at 440 nm (FLUOstar Omega, BMG Labtech, Germany).

*In* *vitro* cytotoxicity of the presented compounds was further determined by the MTT assay in a wide range of human cancer cell lines, i.e. human breast adenocarcinoma (MCF7; ECACC no. 86012803), human osteosarcoma (HOS; ECACC no. 87070202), lung carcinoma (A549; ECACC no. 86012804), malignant melanoma (G-361; ECACC no. 88030401), cervix epitheloid carcinoma (HeLa; ECACC no. 93021013), ovarian carcinoma (A2780; ECACC no. 93112519), *cisplatin*-resistant ovarian carcinoma (A2780R; ECACC no. 93112517) and prostate carcinoma (22Rv1; ECACC no 105092802) cancer cell lines purchased from European Collection of Cell Cultures (ECACC). The cells were cultured according to the ECACC instructions. They were maintained at 37°C and 5% CO_2_ in a humidified incubator. The cells were treated with the complexes **1**–**9**, free HL_1–9_ molecules, HAuCl_4_, AuCl and *cisplatin* (applied up to 50 µM) for 24 h, using multi-well culture plates of 96 wells. In parallel, the cells were treated with vehicle (DMF; 0.1%, v/v) and Triton X-100 (1%, v/v) to evaluate the minimal (i.e. positive control), and maximal (i.e. negative control) cell damage, respectively. The MTT assay was performed spectrophotometrically at 540 nm (TECAN, Schoeller Instruments LLC, Prague, CZ).

### Drug Treatment and Induction of Inflammatory Response

Differentiated macrophages were pretreated with 300 nM solutions of the complexes **1**–**9**, HL_1–9_, AuCl, [AuCl(PPh_3_)], PPh_3_ and Auranofin dissolved in DMSO (the final DMSO concentration was 0.1%) and with 0.1% DMSO solution (vehicle) for 1 h; the given concentrations of the tested compounds lack the cytotoxic effect (cell viability >94%). The inflammatory response was triggered by addition of 1.0 µg/mL LPS dissolved in water to the pretreated macrophages, control cells were without the LPS treatment.

### RNA Isolation and Gene Expression Evaluation

For the evaluation of the expression of TNF-α, IL-1β, and β-actin mRNA, the total RNA was isolated directly from the THP-1 cells in cultivation plates using a RealTime Ready Cell Lysis Kit, according to the manufacturer’s instructions.

The gene expression was quantified by two-step reverse-transcription quantitative (real-time) PCR (RT-qPCR). The reverse transcription step was performed by Transcriptor Universal cDNA Master using cell lysate as a template. The reaction consists of 3 steps: (1) primer annealing, 29°C for 10 min; (2) reverse transcription, 55°C for 10 min; and (3) transcriptase inactivation, 85°C for 5 min. FastStart Universal Probe Master and Gene Expression assays were used for qPCR. These assays contain specific primers and TaqMan probes that bind to an exon-exon junction to avoid DNA contamination. The parameters for the qPCR work were adjusted according to the manufacturer’s recommendations as follows: 50°C for 2 min, then 95°C for 10 min, followed by 40 cycles at 95°C for 15 s and 60°C for 1 min. The results were normalized to the amount of ROX reference dye, and the change in gene expression was determined by the ΔΔC_T_ method. Transcription of the control cells was set as 1 and other experimental groups were multiples of this value.

### Evaluation of Cytokine Secretion by ELISA

Macrophages, which were pretreated with the tested compounds for 1 h, were incubated with LPS for next 24 h. After this period, the medium was collected and the concentration of TNF-α and IL-1β was measured by an Instant ELISA kit according to the manufactures’ manual.

### Determination of IκB Degradation by Western Blot

Macrophage-like THP-1 cells were pretreated with the tested compounds and stimulated by LPS as described above. Thirty minutes after the addition of LPS, the medium was aspirated and cells were washed by cold PBS. Subsequently, the cells were collected using the lysis buffer [50 mM Tris-HCl pH 7.5, 1 mM EGTA, 1 mM EDTA, 1 mM sodium orthovanadate, 50 mM sodium fluoride, 5 mM sodium pyrophosphate, 270 mM sucrose, 0.1% (v/v) Triton X-100, and cOmplete Protease Inhibitor Cocktail (Roche, Germany)] and scraper. The lysis of cells was facilitated by short (ca. 30 s) incubation in the ultrasonic water bath. The protein concentration was determined according to Bradford’s method. For protein separation, 30 µg of protein was loaded onto 12% polyacrylamide gel. Then, they were electrophoretically transferred on the PVDF membranes, which were subsequently blocked by 5% BSA dissolved in TBST buffer [150 mM NaCl, 10 mM Tris base pH 7.5, 0.1% (v/v) Tween-20]. The membranes were incubated with the primary anti-IκB-α antibody at the concentration of 1∶500, or with the primary anti-β-actin at the concentration of 1∶5000 at 4°C for 16 h. After washing, the secondary anti-mouse IgG antibody diluted 1∶2000 was applied on the membranes and incubated for 1 h at laboratory temperature (∼22°C). The amount of the bound secondary antibody was detected colorimetrically by an Opti-4CN kit according to the manufacturer’s manual.

### Statistical Evaluation

The cytotoxicity data were expressed as the percentage of viability, when 100% represent the treatment with vehicle (DMF or DMSO). The experiments were conducted in triplicate using cells from different passages. The IC_50_ values were calculated from viability curves. The results are presented as arithmetic mean ± standard error of the mean (SE). The significance of the differences between the results was evaluated by the ANOVA analysis with p<0.05 considered to be significant (QC Expert 3.2, Statistical software, TriloByte Ltd., Pardubice, CZ).

The statistically significant differences between individual groups during anti-inflammatory activity testing were assessed by the one-way ANOVA test, followed by Tukey’s *post-hoc* test for multiple comparisons. GraphPad Prism 5.02 (GraphPad Software Inc., San Diego, CA, USA) was used for the analysis.

### Interactions with L-Cysteine and Reduced Glutathione Analyzed by Mass Spectrometry

The interaction experiments between the selected representative complexes **1** and **6**, bearing the same ethoxy-substitution on the C6 atom, but differing in the substituents in the C2 position, and the mixture of physiological levels of cysteine and glutathione were performed on a Thermo Scientific LTQ Fleet Ion-Trap mass spectrometer, using the positive ionization mode. The reaction system contained the physiological concentrations of L-cysteine (290 µM) and reduced glutathione (6 µM) [36] and the tested complex (20 µM) in the methanol:water (1∶1, v/v) mixture. The reference system was comprised of the solution of complex (20 µM) in the methanol:water (1∶1, v/v) mixture. The flow injection analysis (FIA) method was used to introduce the reaction system (5 µL spikes) into the mass spectrometer, while pure acetonitrile was used as a mobile phase. The ESI-source was set up as follows: source voltage was 4.5 kV, the vaporizer temperature was 160°C, the capillary temperature was 275°C, the sheath gas flow rate was 30 L/min, and auxiliary gas flow rate was 10 L/min. The system was calibrated according to the manufacturer specifications and no further tuning was needed.

## Results and Discussion

### General Properties of the Au(I) complexes

This work reports on the preparation, thorough characterization, and *in* *vitro* cytotoxic and anti-inflammatory activities of a series of gold(I)-triphenylphosphine complexes [Au(L_1–9_)(PPh_3_)] involving mono- or disubstituted derivatives of hypoxanthine (HL_1–9_). The complexes were synthesized by a modification of a previously published method [15], [16], i.e. by a reaction of the precursor complex [AuCl(PPh_3_)] with an equimolar amount of the corresponding organic molecule HL_1–9_ in acetone with the addition of 1 M NaOH. The prepared complexes **1**–**9** are very well soluble in acetone, alcoholic solvents, toluene, DMSO and DMF, and partially soluble in water at laboratory temperature. The complexes were prepared non-solvated, as evidenced by the results of simultaneous TG/DTA analysis (see Figure S3 in File S1). The complexes were further characterized as chemical individuals by elemental analysis, ^1^H, ^13^C and ^31^P NMR spectroscopy, FT-IR spectroscopy and ESI–MS. Single crystals suitable for X-ray analysis were prepared in the cases of **1** and **3**, thus providing information about the molecular and crystal structures of the complexes (discussed in detail below).

^1^H and ^13^C NMR spectra were measured for all the presented complexes. The obtained results were very beneficial for the confirmation of the purity and composition of **1**–**9**. The observed signals unambiguously proved the presence of both hypoxanthine and phosphine derivatives in the structures of the Au(I) complexes as well as gave information about the coordination mode of these ligands. The most conclusive evidence concerning the identification of the donor atom of HL_1–9_ was obtained from the ^13^C NMR spectra by comparing the chemical shifts in the uncoordinated and coordinated hypoxanthine derivative. The resulting differences, calculated as coordination shifts; *Δδ* = *δ_complex_*–*δ_ligand_*, ppm; Table 1), showed that the greatest changes occurred for the signals corresponding to the carbons C4 and C8, shifted by 2.02–4.82 ppm, and 3.57–6.89 ppm, respectively. This observation indirectly pointed to N9 as the coordination site, as the two mentioned carbons lie in the direct vicinity to this nitrogen. The set of the most intensive signals at around 130 ppm, characteristic of all the spectra of **1**–**9**, was assigned to the carbon atoms of triphenylphosphine. Accordingly, the ^1^H NMR spectra of all the nine Au(I) complexes showed very strong multiplet signals at around 7.70 ppm with the relative integral intensity well corresponding to the calculated value of 15 hydrogens of PPh_3_. Additionally, the C8 H signal of the corresponding HL_1–9_ ligand was shifted by 0.08–0.27 ppm upfield in the spectra of the complexes with respect to the spectra of the uncoordinated HL_1–9_, corresponding with the results following from ^13^C NMR spectroscopy about N9 being the site of coordination to gold. What should be also pointed out is the absence of the signal assignable to the proton N9 H in the spectra of **1**–**9**, which well agrees with the presence of deprotonated hypoxanthine-based ligands in the studied complexes symbolized as L_1–9_. All the ^31^P NMR spectra of the complexes were characteristic of the presence of one singlet at 31.67–33.13 ppm (see Figure S5 in File S1), which is significantly shifted as compared to the signal of free PPh_3_ (–5.96 ppm), which confirms the coordination of PPh_3_ to gold(I) atom through phosphorus, thus forming the Au–P bond. In summary, the presence and chemical shift of all the detected signals in ^1^H, ^13^C and ^31^P NMR spectra, as well as the relative intensity of the peaks in the proton spectra, indicate that the gold(I) atom in **1**–**9** is coordinated by one deprotonated hypoxanthine-derived compound (L_1–9_) binding via N9 and one PPh_3_ molecule. These conclusions are in good agreement with the results following from single crystal X-ray analysis of complexes **1** and **3** discussed below.

**Table 1:** ^1^H and ^13^C NMR coordination shifts (Δδ = δ_complex_–δ_ligand_; ppm) of calculated for **1**–**9**.

	^1^H NMR		^13^C NMR				
	C2 H	C8 H	C2	C4	C5	C6	C8
**1**	–0.05	–0.08	–0.90	4.80	–1.14	–1.04	4.40
**2**	–0.05	–0.10	–0.60	4.82	–0.59	–0.21	3.57
**3**	–0.11	–0.23	0.98	2.20	–0.56	–0.24	5.11
**4**	–0.13	–0.23	0.86	3.00	–0.81	0.50	5.03
**5**	–0.12	–0.25	–0.01	2.16	–1.29	–0.32	5.85
**6**	n.a.	–0.27	–0.16	2.02	–1.17	0.05	6.02
**7**	n.a.	–0.10	–0.04	4.01	–0.54	–0.73	6.89
**8**	n.a.	–0.12	–0.49	2.38	–0.74	–0.35	4.89
**9**	n.a.	–0.24	–0.53	3.22	–1.01	–0.18	5.70

n.a. – not available.

Mass spectra measured in the positive ionization mode also indirectly confirmed the composition of the presented complexes, as all the spectra of **1**–**9** contained the [M+H]^+^ molecular peaks. The presence of the hypoxanthine-based derivatives was demonstrated by the observed peaks corresponding to the adducts of [HL_n_+Na/K]^+^ (for details of ESI+MS characterization, see File S1).

Further properties of the presented Au(I) complexes were studied by FT-IR spectroscopy. The courses of the spectra of **1**–**9** in the mid-IR region were qualitatively very similar to those of the uncoordinated HL_1–9_. The very intensive peaks at 1600–1578 cm^−1^ could be assigned to the ν(C–^…^N)_ring_ stretching vibrations characteristic of heterocyclic molecules with nitrogen ring atoms. Further assigned characteristic vibrations could be found at 3057–3048 cm^−1^ for v(C-H)_ar_, 2986–2889 cm^−1^ for v(C-H)_aliph_, and also 1480–1436 cm^−1^ for ν(C–^…^C)_ring_. The typical vibrations of the ether functional group found in the *O*6-substituted hypoxanthine derivatives were detected at 1339–1272 cm^−1^ for the aromatic C6–O stretch and at 1118–1087 cm^−1^ for the O–C10 stretch. The medium to strong intensity bands found between 800–700 cm^−1^, and at ca. 690 cm^−1^ can be attributed to the out-of-plane C–H bending vibrations, and ring bending, respectively, determining the presence of an aromatic ring in all the complexes **1**–**9** [37], [38]. In the far spectra of the Au(I) complexes, new bands were identified as compared with the spectra of free HL_1–9_. These maxima at 506–492 cm^−1^, and 329–321 cm^−1^ could be assigned to v(Au–N), and v(Au–P) stretching vibrations, respectively, although it should be noted that triphenylphosphine alone has intensive vibrations in this region of IR spectra, so these bands could be overlapped [15], [16], [37], [39].

### Molecular and Crystal Structures of [Au(L_1_)(PPh_3_)](1) and [Au(L_3_)(PPh_3_)] (3)

**Figure 3:**
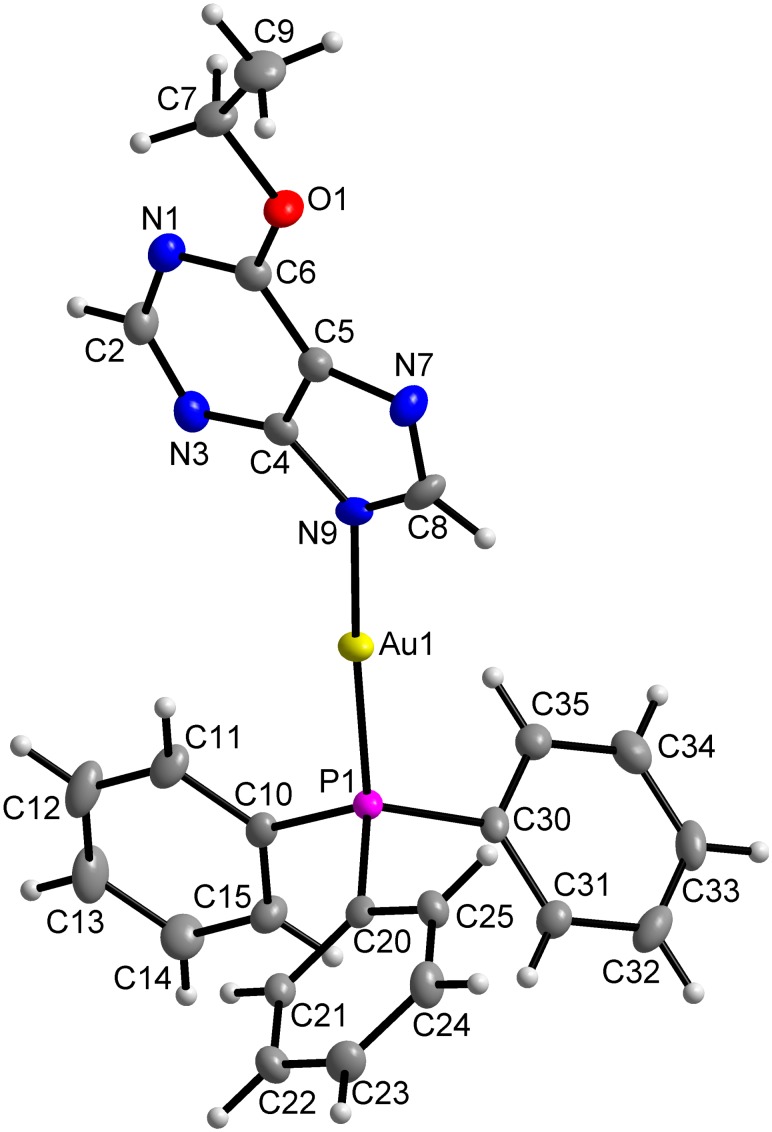
Molecular structure of [Au(L_1_)(PPh_3_)] (1). The molecular structure of **1** showing the atom numbering scheme. Non-hydrogen atoms are displayed as ellipsoids at the 50% probability level.

**Figure 4:**
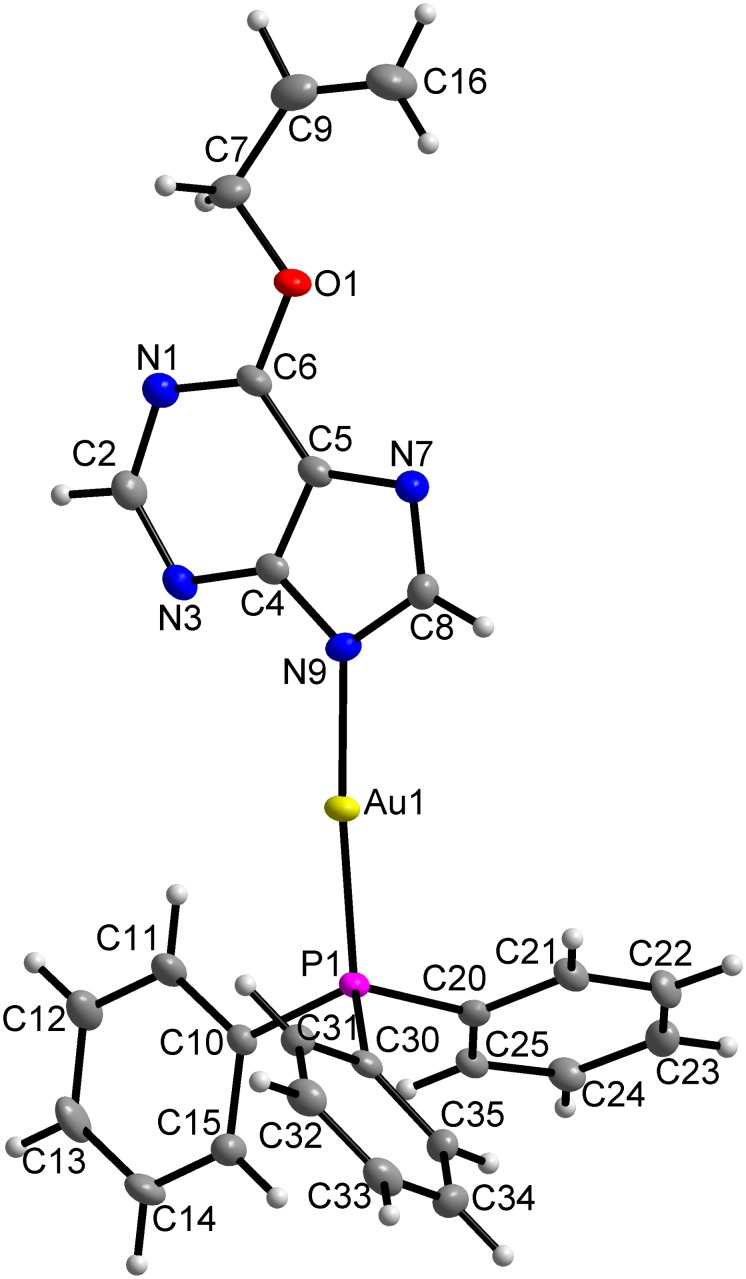
Molecular structure of [Au(L_3_)(PPh_3_)] (3). The molecular structure of **3** showing the atom numbering scheme. Non-hydrogen atoms are displayed as ellipsoids at the 50% probability level.

The crystallization method of slow *n*-hexane diffusion into the saturated toluene solution of the complexes allowed the preparation of single crystals suitable for X-ray analysis of two complexes, [Au(L_1_)(PPh_3_)](**1**) and [Au(L_3_)(PPh_3_)] (**3**), where HL_1_ = 6-ethoxy-9*H*-purine and HL_3_ = 6-allyloxy-9*H*-purine. The molecular structures are depicted in Figure 3, and 4, respectively. Crystal data and structure refinement parameters (Table S1 in File S1), selected bond lengths and angles (Table S2 in File S1), as well as non-covalent interaction parameters (Tables S3 and S4 in File S1) are given in File S1.

In both complexes **1** and **3**, the central gold(I) atom is two-coordinated in the distorted linear arrangement of the {NP} donor set formed by one *N*-donor deprotonated hypoxanthine derivative and the electroneutral molecule of triphenylphosphine bonded via phosphorus. Comparing the coordination bonds in **1** and **3**, both bond lengths are mutually comparable between the complexes, as they were found to be 2.046(3), and 2.042(3) Å for Au–N; and 2.2344(9), and 2.2356(8) Å for Au–P, respectively. On the other hand, it is evident that the Au–P bonds are significantly longer than the Au–N bonds, which is most likely connected with the bulky phenyl moieties surrounding the phosphorus donor atom in PPh_3_. This bond distance difference is not uncommon in similar complexes. The search in the Cambridge Structural Database (CSD ver. 5.35, February 2014 update) [40] within the deposited 113 mononuclear compounds involving the N-Au-PPh_3_ motif with the central gold atom in the linear geometry proved that the Au–N and Au–P bond lengths in **1** and **3** fall within the interval of such bond lengths found in the above specified set of compounds, i.e. 1.971–2.134 Å, and 2.22–2.278 Å, respectively. The distortion of the linear geometry is well demonstrated on the dimensions of the N9–Au1–P1 angles, which are equal to 172.73(10)° in **1** and 175.65(8)° in **3**. These values also clearly reveal that the distortion from the ideal angle of 180° is significantly higher in complex **1** than in **3**. This difference could most likely be connected with a somewhat different array of non-covalent contacts found in the complexes. In general, the crystal structures of both complexes **1** and **3** are characterised by absence of strong non-covalent contacts, such as typical hydrogen bonds. The crystal structures of both the complexes are stabilized by C–H···C, C–H···N and C–H···O non-covalent contacts. For more details, see Supplementary Information, Tables S3 and S4 in File S1, Figures S1 and S2 in File S1.

### *In Vitro* Cytotoxicity

For the evaluation of *in* *vitro* cytotoxicity, the presented complexes were tested by the MTT assay and the results were compared with the clinically applied metallodrug *cisplatin*, used as the reference standard, as well as with the starting compounds, i.e. hypoxanthine derivatives HL_1–9_, and gold-containing inorganic compounds AuCl or HAuCl_4_ (applied in the concentration range of 0.01–50.0 µM, unless their solubility was found to be lower). The first phase of testing involved screening of all the prepared complexes **1**–**9** against two human cancer cell lines, breast adenocarcinoma (MCF7) and osteosarcoma (HOS). The starting compounds were found inactive up to 50 µM or to the limiting concentration of their solubility against these cells. Then, this testing showed a varied effect of the tested complexes on the viability of the selected cancer cell lines. Complex **7** showed to be inactive up to the tested concentration range on both cell lines (IC_50_>50 µM). Complexes **8** and **9** were found to be cytotoxic inactive against the MCF7 cells in the used concentration ranges given by their solubility, therefore their cytotoxicity can be evaluated as >25 µM. Complex **2** was moderately active on both cell lines, however, the IC_50_ values, i.e. 27.1±0.2 µM (MCF7) and 30.0±2.1 µM (HOS), were higher than those for *cisplatin* (in the case of MCF7, significantly higher, ANOVA, p<0.05). This pointed to the fact that the *n*-butyl group as the R1 substituent showed to be the least beneficial for the resulting cytotoxic activity against these cell lines within the tested series of compounds (*n*-Bu present in **2** and **7**). The best results were observed for the complexes **4**–**6**, all of which possessed significantly better cytotoxicity than *cisplatin* (ANOVA, *p*<0.05) against both cell lines, concretely the IC_50_ values were found to be 2–5-times lower. The most promising data were found for complex **4** against the MCF7 cell line, i.e. IC_50_ = 3.7±0.5 µM (17.9±1.2 µM for *cisplatin*), and for complex **6** against HOS cells, i.e. 4.0±0.3 µM as compared with 20.5±0.1 µM for *cisplatin* (Table 2).

**Table 2:** *In vitro* cytotoxicity of complexes 1–9 and *cisplatin* against MCF7 and HOS cancer cell lines.

	Cell Line		
Compound	MCF7	HOS	THP-1
**1**	15.22±0.78	19.20±0.71	1.17±0.04
**2**	27.10±0.21	30.02±2.13	1.03±0.04
**3**	12.40±1.07	15.60±1.10	1.87±0.09
**4**	3.66±0.48	11.30±0.67	2.15±0.19
**5**	6.30±0.80	6.56±0.97	1.89±0.11
**6**	5.23±0.68	3.96±0.29	1.94±0.12
**7**	>50	>50	1.97±0.14
**8**	>25	18.80±0.70	5.28±0.21
**9**	>25	16.80±0.27	>10
*cisplatin*	17.90±1.17	20.50±0.10	-
*Auranofin*	1.10±0.30^a^	n.d.	0.88±0.04

The results of the *in* *vitro* cytotoxic activity testing of **1**–**9** and *cisplatin* against human breast adenocarcinoma (MCF7) and osteosarcoma (HOS): cells were treated with the tested compounds for 24 h; measurements were performed in triplicate, and cytotoxicity experiment was repeated in three different cell passages; data are expressed as IC_50_±SE (µM).

a adopted from Ref. [42]; n.d. – not determined.

Before using the THP-1 cell line for testing of the anti-phlogistic effect, cytotoxicity of complexes **1**–**9** and uncoordinated hypoxanthine derivatives HL_1–9_ was also tested on this cell line (Table 2). The IC_50_ values for **1**–**7** were between 1.03–2.15 µM, which are significantly lower than for the MCF7 and HOS cell lines. Interestingly, **8** has the IC_50_ only 5.3±0.2 µM and **9** does not even have any cytotoxic effect up to the concentration of 10 µM, whereas their structural analogues without chlorine at C2 (**3** and **4**) have the IC_50_ values equal to 1.87±0.09 µM, and 2.15±0.19 µM, respectively. In all the cases, cytotoxicity was lower (i.e. the IC_50_ values were higher; 1.03–5.28 µM) than for commercially used drug Auranofin (IC_50_ = 0.88±0.04 µM). Recently, the gold(I) complexes with the general composition [Au(PPh_3_)(Y)], where Y represents an *S*-coordinated thioamide ligand, showing remarkable cytotoxicity towards leiomyosarcoma cells with the IC_50_ values in the comparable range as **1**–**7** (0.7–2.1 µM) have been reported [41]. Complexes **1**–**8** additionally demonstrate the hormesis effect (higher viability; in this case up to 168%) around the concentration of 0.3 µM.

**Figure 5:**
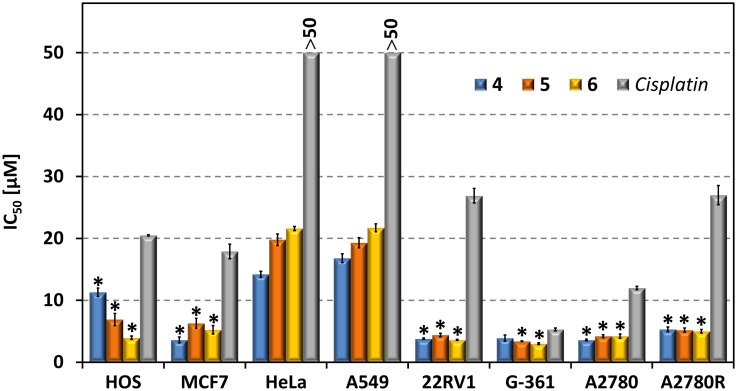
The results of *in* ***vitro*** **cytotoxicity against selected cancer cell lines for complexes 4–6 and** ***cisplatin*** **.** The cells were exposed to the employed complexes for 24 h. Measurements were performed in triplicate and each cytotoxicity experiment was repeated three times. The given IC_50_±SE (µM) values represent an arithmetic mean. The asterisk (*) denotes significant difference (ANOVA, p<0.05) between **4**–**6** and *cisplatin*.

Based on the promising results for complexes **4**–**6** against all the three above mentioned cell lines, further testing was performed for these representatives on a panel of human cancer cell lines involving lung carcinoma (A549), malignant melanoma (G361), cervix epitheloid carcinoma (HeLa), ovarian carcinoma (A2780), ovarian carcinoma resistant to *cisplatin* (A2780R) and prostate carcinoma (22Rv1). The testing again revealed that the starting compounds (HL_n_ and AuCl, HAuCl_4_) were not toxic in the tested concentration range, only a weak antiproliferative effect was observed for HAuCl_4_ on the G361 cell line (38.1±0.8 µM). The IC_50_ values determined for **4**–**6** showed that viability of all the employed cell lines was reduced by the tested complexes comparably or better than by the metallodrug *cisplatin* (Table 3, Figure 5). Complexes **5** and **6** were significantly more *in* *vitro* antitumour active (ANOVA, *p*<0.05) against all the cell lines as compared with *cisplatin*, which is valid also for complex **4** with the exception of the G361 cells, against which its activity was found comparable with *cisplatin* (3.9±0.5 µM, and 5.3±0.2 µM, respectively). Focusing on individual cell lines, the lowest antiproliferative effect of **4**–**6** was observed for A549 and HeLa, which in the testing did not respond to the treatment by *cisplatin* up to 50 µM. The IC_50_ values for the tested Au(I) complexes showed moderate activity in the micromolar range (≈15–20 µM). On the other hand, low micromolar IC_50_ values (≈3–5 µM) resulted from the cytotoxicity testing against all the other cancer cell lines, *i.e.* G361, 22Rv1, A2780 as well as A2780R. It should be also pointed out that complexes **4**–**6** showed to be ca. 3-times (A2780), 5-times (A2780R) and 7-times (22Rv1) more effective than *cisplatin*. Moreover, the evaluation of cytotoxicity of **4**–**6** on both A2780 cell lines sensitive and resistant to *cisplatin* allowed the calculation of the resistance factors (RF), *i.e.* the ratio of IC_50_(A2780R)/IC_50_(A2780). The RF values are equal to 1.19 (**4**), 1.14 (**5**), 1.21 (**6**) and 2.25 (*cisplatin*), which unambiguously shows that **4**–**6** are able to circumvent the acquired resistance of cancer cells to *cisplatin*. It can be additionally pointed out that the tested complexes are comparably active on both A2780 and A2780R cell lines, as there is no statistically significant difference in their cytotoxic activity against these cancer cells (ANOVA, *p*<0.001).

**Table 3:** *In* *vitro* cytotoxicity of complexes 4–6 and *cisplatin* against a panel of cancer cell lines.

	Cell Line					
Compound	A549	G-361	HeLa	A2780	A2780R	22Rv1
**4**	16.8±0.7	3.9±0.5	14.2±0.5	3.6±0.1	4.3±0.2	3.8±0.1
**5**	19.3±0.8	3.4±0.1	19.8±0.9	4.2±0.2	4.8±0.6	4.4±0.3
**6**	21.7±0.4	3.0±0.1	21.6±0.3	4.2±0.3	5.1±0.4	3.6±0.1
*cisplatin*	>50	5.3±0.2	>50	12.0±0.3	27.0±1.5	26.9±1.2

The results of the *in* *vitro* cytotoxic activity testing of **4**–**6** and *cisplatin* against the human cancer cell lines. Cells were treated with the tested compounds for 24 h; measurements were performed in triplicate, and cytotoxicity experiments were repeated in three different cell passages; data are expressed as IC_50_±SE (µM).

### *In Vitro* Anti-Inflammatory Activity

**Figure 6:**
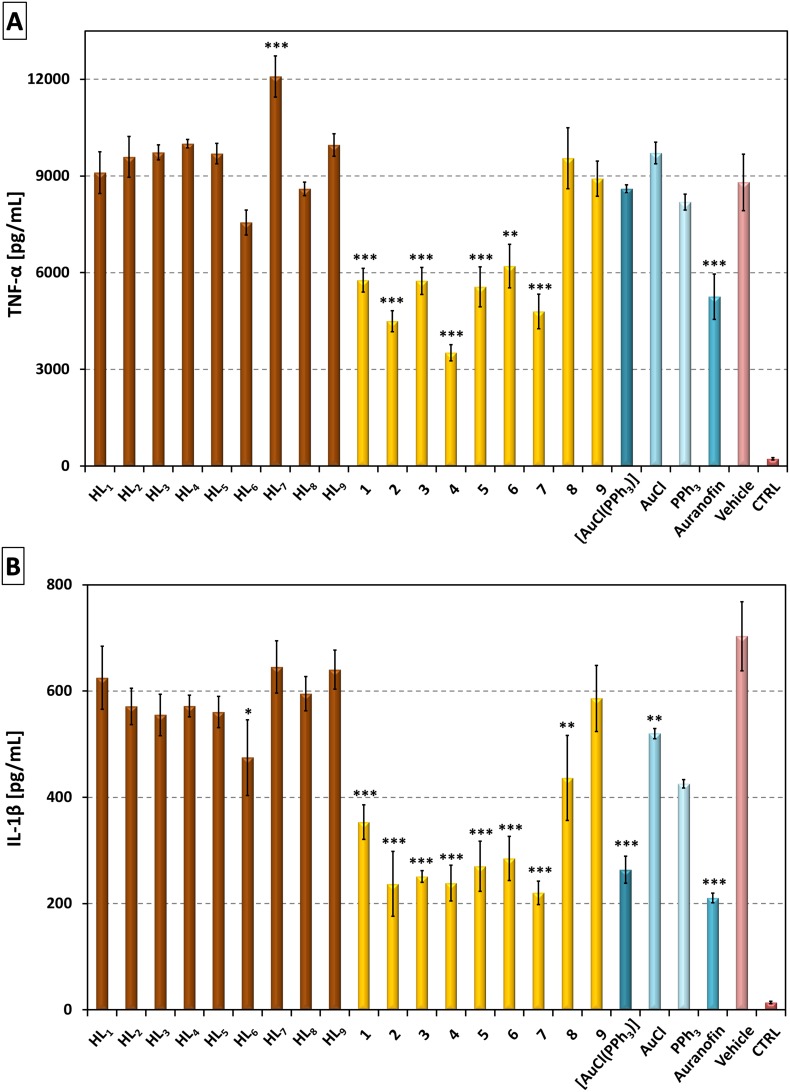
Effects of the Au(I) complexes 1–9, reference drug Auranofin, [AuCl(PPh_3_)], AuCl, PPh_3_ and HL_1–9_ on LPS-induced TNF-α (A) and IL-1β (B) secretion. The cells were pretreated with the tested compounds (300 nM) or the vehicle (0.1% DMSO) only. After 1 h of the incubation, the inflammatory response was induced by LPS [except for the control cells (CTRL)]. The secretion was determined 24 h after the LPS addition. The results are expressed as means±SE of three independent experiments. Significant difference in comparison to: *vehicle-treated cells (p<0.05), **vehicle-treated cells (p<0.01), ***vehicle-treated cells (p<0.001).

To evaluate the anti-inflammatory effect of the presented complexes *in* *vitro*, their ability to diminish the production of pro-inflammatory cytokines TNF-α and IL-1β in LPS-stimulated macrophages derived from the THP-1 cell line was determined. The results of this study showed that complexes **1**–**7** significantly decreased secretion of TNF-α in the LSP-stimulated cells, while complexes **8** and **9** exhibited no effect, as the extent of their influence on TNF-α secretion was comparable to the vehicle alone (0.1% DMSO) (Figure 6A). It is interesting to note, that the comparison of complexes **1**–**4** and their analogues **6**–**9** differing only in the substitution at C2 (hydrogen, and chlorine, respectively) showed that in all the cases, the complexes **1**–**4** were more effective in the TNF-α level attenuation. Moreover, all the active complexes (**1**–**7**) had comparable activity as the commercial reference drug Auranofin, but importantly they possessed a lower cytotoxic effect in the employed THP-1 cell line. To elucidate the role of each constituent part of the complexes (*N*-donor ligand, PPh_3_ or gold(I) species) in diminishing TNF-α secretion, free hypoxanthine derivatives (HL_1–9_), [AuCl(PPh_3_)], PPh_3_, and AuCl were also tested. It was found that none of these starting materials showed the anticipated effect. Moreover, the compound HL_7_ significantly increased the production of TNF-α. These results indicated that in order to show the anti-inflammatory effect, the corresponding ligand has to be bonded into the Au(I)-complex. This observation is in agreement with the previous studies, where no anti-phlogistic effect was observed for free and uncoordinated purine-derived compounds applied as ligands in Au(III)-complexes [28].

The extent of the influence of complexes **1**–**9** on IL-1β secretion was very similar as for TNF-α (Figure 6B), but it should be noted that IL-1β production was affected slightly more than TNF-α. The same observation was also made in previous studies [15], [43], which showed that the tested gold(I) complexes preferentially inhibit IL-1β production as compared to TNF-α. The results proved that similarly to TNF-*α*, compounds **1**–**7** significantly decreased the level of IL-1β, whereas complex **9** showed again no effect. The only difference as compared to the above-described TNF-α production influence study was found for complex **8**, which was inactive in the case of TNF-α secretion, but it significantly reduced the production of IL-1β. However, its inhibitory activity was still lower as compared with the activity of **1**–**7**. Testing of the constituent parts of the complexes as well as of starting materials revealed the element necessary for diminishing the production of IL-1β. The free HL_n_ molecules were again found to be inactive, with the exception of HL_6_ which reduced the level of the cytokine significantly as compared with the vehicle-treated cells (*p*<0.05). On the other hand, unlike in the case of TNF-α, AuCl significantly decreased the level of this pro-inflammatory cytokine (*p*<0.01) as well as the [AuCl(PPh_3_)] complex, which even curtailed its level as effectively as complexes **1**–**9**. These results indicate that the presence of the {Au-PPh_3_} moiety in the complexes is crucial for the reduction of IL-1β secretion, but the coordinated *N*-donor organic ligand can dramatically change the activity of such a complex as it is visible on the example of the complexes **8** and **9** with low or no anti-inflammatory activity.

**Figure 7:**
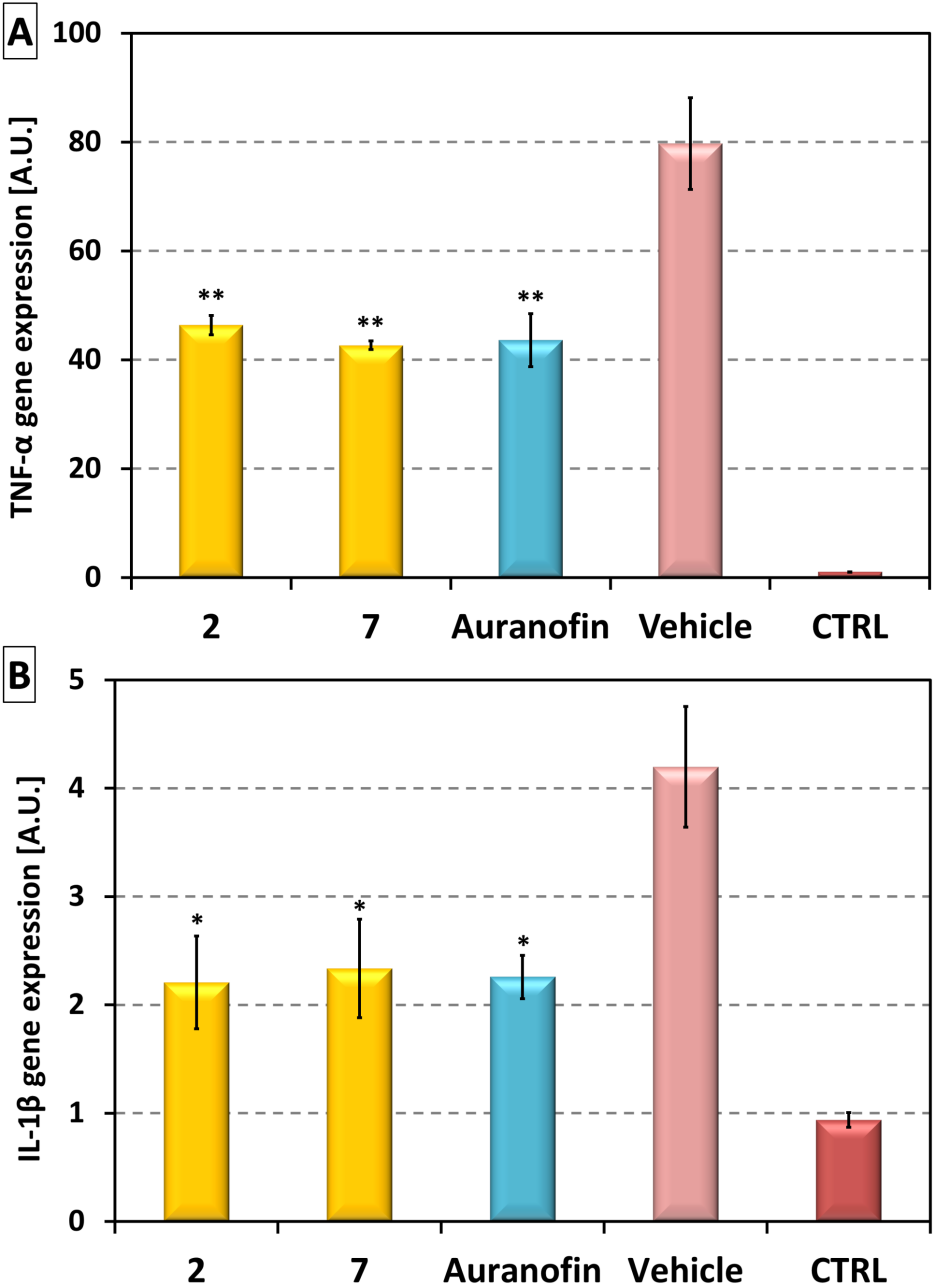
Effects of the Au(I) complexes 2 and 7, and reference drug Auranofin on gene expression of TNF-α (A) and IL-1β (B). THP-1 macrophages were pretreated with complexes **2**, **7** and Auranofin at the concentration of 300 nM or the vehicle (0.1% DMSO) only. After 1 h of the incubation, the inflammatory response was induced by LPS [except for the control cells (CTRL)]. After 2 h, the level of TNF-α and IL-1β mRNA was evaluated by RT-qPCR. The amount of cytokine mRNA was normalised to β-actin mRNA. The results are expressed as means±SE of three independent experiments. A.U.  = arbitrary unit. Significant difference in comparison to: *vehicle-treated cells (p<0.05), **vehicle-treated cells (p<0.01).

To determine whether the production of the pro-inflammatory cytokines TNF-α and IL-1β is regulated by a post-translation or post-transcription mechanism in the presence of the Au(I) complexes, the level of the corresponding mRNA was determined. The obtained results showed that selected representative complexes **2** and **7** were able to decrease the transcription of these cytokines after the LPS stimulation (Figure 7). This finding indicates that the target site for the Au(I) complexes should be up-stream the transcription, which is in accordance with previous studies [15], [44].

The transcription of the cytokines TNF-α and IL-1β is controlled by transcription factor NF-κB. This protein is kept in cytoplasm by its inhibitor IκB. After the activation of the signalling pathway, this inhibitor is degraded [45]. Gold-containing compounds are known for their ability to inhibit this signalling pathway [46]. The results from the herein presented study are in agreement with the suggested mechanism. Complexes **2** and **7**, as well as Auranofin, were able to attenuate the degradation of IκB nonsignificantly (Figure S4 in File S1). This blocking of the NF-κB signalling pathway leads to lower transcription of TNF-α and IL-1β genes and thus, decreases their secretion.

### Interactions with a Mixture of Cysteine and Glutathione Analysed by ESI-MS

As soft Lewis acids, Au(I) species prefer the formation of strong coordination bonds with soft Lewis base ligands, i.e. thiolate or selenolate ions, or phosphine derivatives, while the latter ones form the most stable bonds. It is a known fact, that Au(I) complexes behave likewise, as they bind to sulfanyl-containing substances, such as amino acid cysteine (Cys) or small proteins, such as glutathione (GSH), and with high molecular weight proteins (*e.g.* albumin or globulins [47]), in the biologically relevant environments (*i.e.* blood or serum) by the ligand exchange mechanism. The complexes formed this way can be considered as transport intermediates. The exchange of *N*-ligands for *S*-ligands occurs relatively quickly (within 20 minutes when interacting with albumin and globulins in the blood [48]), while the *P*-ligand exchange proceeds much more slowly. It seems that in this mechanism the cooperative effects of adjacent thiolato or selenolato ligands in the neighbourhood of the interaction site play an important role. In connection with the above mentioned, the ligand exchange is interpreted as one of the molecular mechanisms of incorporation of gold into the active site of selenium-containing flavoreductases, such as thioredoxin reductase [49]. In the scope of our work, we strived to uncover the molecular behaviour of selected complexes **1** and **6** (applied in the concentration of 20 µM, which approximately corresponds to the highest therapeutic blood levels of gold during chrysotherapy [50]) in biologically relevant conditions using a mixture of cysteine (at the 290 µM concentration) and reduced glutathione (at the 6 µM concentration) [36].

**Figure 8:**
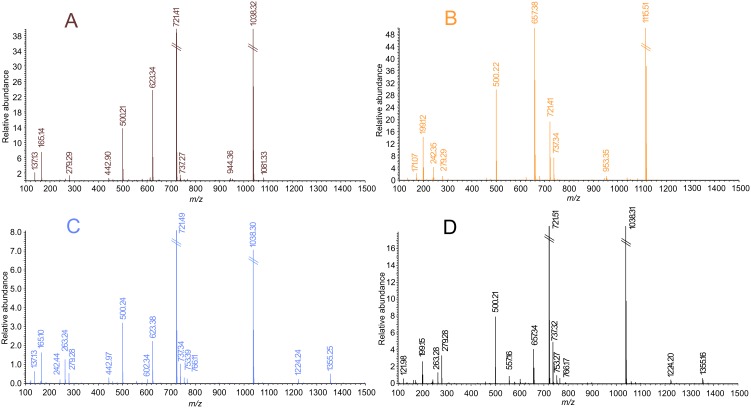
The results of the ESI-MS study of complex 1 (A) and 6 (B) solutions and interacting systems involving the mixture of physiological levels of cysteine and reduced glutathione and complex 1 (C) or complex 6 (D).

Based on the results of the ESI-MS experiments, we confirmed that both complexes **1** and **6** react with the used sulfhydryl-containing substances in time-independent manner by the ligand-exchange mechanism based on the substitution of the *N*-ligand (L_n_) by the cysteine or glutathione molecule. This mechanism was confirmed by the emergence of ions at 1224.24 *m/z*, corresponding to the [(Au-PPh_3_)_2_+GSH]^+^ intermediate, and an ion at 1355.25 *m/z*, corresponding to the ionic species [Cys+(Au-PPh_3_)_2_+PPh_3_+CH_3_OH+Na]^+^ (see Figure 8). Unlike our previous reports on the biological activities of Au(I) complexes [15], [16], in this interaction experiments we observed both the intermediates involving cysteine and glutathione molecules. This might indicate that the herein reported compounds are much more susceptible to the sulfur-containing molecules with several donor atoms, like glutathione.

In concordance with the above mentioned suggestion as well as in accordance with the previously reported behaviour of some Au(I) complexes in water-containing solutions [16], the mass spectra of the reacting systems involving sulfur-containing molecules and also the reference solutions of complexes revealed a considerable instability of the complexes demonstrated by the appearance of the intensive ion at 721.41 *m/z*, corresponding to the [Au(PPh_3_)_2_]^+^ intermediate, and other ionic species involving the residue Au-PPh_3_ (i.e. [M+(Au-PPh_3_)]^+^; 1081.33 *m/z* for complex **1**, and 1115.51 *m/z* for complex **6**), the free HL_n_ molecules ([HL_1_+H]^+^ at 165.10 *m/z* and ([HL_6_+H]^+^ at 199.12 *m/z*), or the free triphenylphosphine residue (i.e. [PPh_3_+H]^+^ at 263.24 *m/z* and [Au+(PPh_3_)_3_+CH_3_OH+Na]^+^ at 1038.30 *m/z*).

## Conclusions

A series of gold(I) complexes of the general composition [Au(L_n_)(PPh_3_)] (**1**–**9**), involving a combination of *N*-donor (HL_n_) and *P*-donor triphenylphosphine ligands, was prepared and thoroughly characterized. The *in* *vitro* cytotoxicity results against a panel of nine human cancer cell lines (MCF7, HOS, A549, HeLa, A2780, A2780R, 22Rv1, G-361 and THP-1) revealed the complexes as more anticancer active than *cisplatin*, with the best IC_50_ ≈ 1–5 µM. Moreover, the calculated resistance factors, i.e. the ratio of the IC_50_ values found for the *cisplatin* resistant and sensitive cell lines (A2780R)/IC_50_(A2780) showed that the complexes are able to circumvent the acquired resistance of cancer cells to *cisplatin*, as the resistance factor values are equal to 1.19 (**4**), 1.14 (**5**), 1.21 (**6**) as compared to the value of 2.25 for *cisplatin*. Further, the complexes **1**–**9** were evaluated for their *in* *vitro* anti-inflammatory activity on the model of the LPS-activated THP-1 monocytes. It was found out that the complexes **1**–**7** exhibited the ability to influence the cell-cycle of THP-1 cells resulting in the hormetic effect, and they were also able to significantly attenuate the production of pro-inflammatory cytokines TNF-α and IL-1β at comparable levels as gold(I)-based reference drug Auranofin, but with lower toxicity than this metallodrug. Based on the results of the ESI-MS experiments may be concluded that representative complexes **1** and **6** react with the sulfur-containing substances (cysteine and reduced glutathione) in time-independent manner by the ligand-exchange mechanism based on the substitution of the *N*-ligand (L_n_) by the cysteine or glutathione molecule.

## Supporting Information

File S1 **Supporting Information.** The results of elemental analysis, FTIR, ^1^H and ^13^C NMR, and ESI–MS experiments for **1**–**9**. **Table S1.** Crystal data and structure refinements for [Au(L1)(PPh3)] (**1**) and [Au(L3)(PPh3)] (**3**). **Table S2.** Selected bond lengths and angles in complexes **1** and **3. Figure S1.** Parts of the crystal structure of complex [Au(L_1_)(PPh_3_)] (**1**). **Table S3.** Selected non-covalent contacts and their parameters for **1**. **Figure S2.** Parts of the crystal structure of complex [Au(L_3_)(PPh_3_)] (**3**). **Table S4.** Selected non-covalent contacts and their parameters for **3**. **Figure S3.** TG/DTA curves of the complexes **1** and **4**. **Figure S4.** Effects of the Au(I) complexes, and Auranofin on the LPS-induced degradation of IκB-α. **Figure S5.** ^31^P NMR spectrum of complex **6**. (DOCX) CCDC Nos. 1010556 and 1010557 contain the supplementary crystallographic data for **1**, and **3**, respectively. These data can be obtained free of charge via http://www.ccdc.cam.ac.uk/conts/retrieving.html, or from the Cambridge Crystallographic Data Centre, 12 Union Road, Cambridge CB2 1EZ, UK; fax: (44) 1223-336-033; or email: deposit@ccdc.cam.ac.uk.
(DOCX)
