# Supplementary material for: Gold(I)-Triphenylphosphine Complexes with Hypoxanthine-Derived Ligands: In Vitro Evaluations of Anticancer and Anti-Inflammatory Activities
Source: PLoS One. 2014 Sep 16;9(9):e107373. doi: 10.1371/journal.pone.0107373 (PMC4167326; doi:10.1371/journal.pone.0107373)
Supplement: File S1 — Supporting Information. The results of elemental analysis, FTIR, 1H and 13C NMR, and ESI–MS experiments for 1–9. Table S1. Crystal data and structure refinements for [Au(L1)(PPh3)] (1) and [Au(L3)(PPh3)] (3). Table S2. Selected bond lengths and angles in complexes 1 and 3. Figure S1. Parts of the crystal structure of complex [Au(L1)(PPh3)] (1). Table S3. Selected non-covalent contacts and their parameters for 1. Figure S2. Parts of the crystal structure of complex [Au(L3)(PPh3)] (3). Table S4. Selected non-covalent contacts and their parameters for 3. Figure S3. TG/DTA curves of the complexes 1 and 4. Figure S4. Effects of the Au(I) complexes, and Auranofin on the LPS-induced degradation of IκB-α. Figure S5. 31P NMR spectrum of complex 6. (DOCX) CCDC Nos. 1010556 and 1010557 contain the supplementary crystallographic data for 1, and 3, respectively. These data can be obtained free of charge via http://www.ccdc.cam.ac.uk/conts/retrieving.html, or from the Cambridge Crystallographic Data Centre, 12 Union Road, Cambridge CB2 1EZ, UK; fax: (44) 1223-336-033; or email: deposit@ccdc.cam.ac.uk. (DOCX) [file pone.0107373.s001.docx]

**SUPPORTING INFORMATION**

**Gold(I)-Triphenylphosphine Complexes with Hypoxanthine-Derived Ligands: *In Vitro* Evaluations of Cytotoxicity and Anti-Inflammatory Activities**

Radka Křikavová^1^, Jan Hošek^1^, Ján Vančo^1^, Jakub Hutyra^1^, Zdeněk Dvořák^2^, Zdeněk Trávníček^1^*

*^1^ Regional Centre of Advanced Technologies and Materials, Department of Inorganic Chemistry, Faculty of Science, Palacký University, Olomouc, Czech Republic*

*^2^ Regional Centre of Advanced Technologies and Materials, Department of Cell Biology and Genetics, Faculty of Science, Palacký University, Olomouc, Czech Republic*

* Corresponding author: prof. Zdeněk Trávníček; Regional Centre of Advanced Technologies and Materials, Department of Inorganic Chemistry, Faculty of Science, Palacký University, 17. listopadu 12, Olomouc, 771 46, Czech Republic; Tel.: +420 585 634 352; fax: +420 585 634 954; e-mail: [zdenek.travnicek@upol.cz](mailto:zdenek.travnicek@upol.cz)

**Contents:**

**Elemental analysis, FTIR, NMR and ESI–MS data for** **1**-**9**….........................…..……………... Pages S2-S8

**Table S1. Crystal data and structure refinements for [Au(L_1_)(PPh_3_)] and [Au(L_3_)(PPh_3_)]**.......... Page S9

**Table S2. Selected bond lengths and angles in complexes 1 and 3**.…………..........…………….…...Page S10

**Figure S1. Parts of the crystal structure of complex [Au(L_1_)PPh_3_] (1)**….…………………………………Page S11

**Table S3. Selected non-covalent contacts and their parameters for** **1**.……………………………….Page S12

**Figure S2. Parts of the crystal structure of complex [Au(L_3_)PPh_3_] (3)**………………….…………..…….Page S13

**Table S4**. **Selected non-covalent contacts and their parameters for** **3**……………………………….. Page S14

**Figure S3. TG/DTA curves of the complexes 1 and 4**…….…………………………………………………….… Page S15

**Figure S4. Effects of the Au(I) complexes and Auranofin on the degradation of I*κ*B-*α***.......... Page S16

**Figure S5. ^31^P NMR spectrum of complex 6.** ………………………………………………………………………….Page S17

**Elemental analysis, FTIR, NMR and ESI–MS data for** **1**-**9**

[Au(L_1_)(PPh_3_)] (**1**): Yield: 81%, Anal. Calc. for C_25_H_22_N_4_OPAu (HL_1_ = 6-ethoxy-9*H*-purine): C, 48.24%; H, 3.56%; N, 9.00%. Found: C, 47.78%; H, 3.61%; N, 9.44%. ESI+ MS (methanol, m/z) 187 (calc. 187) [HL_1_+Na]^+^, 623 (623) [M+H]^+^. IR (*ν*_ATR_/cm^–1^): 3055m v(C-H)_ar_; 2980m v(C-H)_al_; 2917m; 2848m; 1592s v(C^…^N)_ring_; 1550m; 1477m, 1454m; 1433s v(C^…^C)_ring_; 1377m; 1334m; 1318s v(C_6_-O); 1302s; 1241w; 1196w; 1175m; 1137m; 1101s v(O-C_10_); 1019m; 997w; 970w; 971w; 923w; 890w; 854w; 798w; 747m; 690s; 645m; 585m; 544s v(Au-N); 498s; 448m; 328m v(Au-P). ^1^H NMR, DMF-*d_7_*, δ, ppm: 8.45, 1H, s, HC^2^, 8.36, 1H, s, HC^8^, 7.74-7.68, 15H, m, HC^PPh3^, 4.63, 2H, q, HC^10^, 1.41, 3H, t, HC^11^. ^13^C NMR, DMF-*d_7_*, δ, ppm: 159.59 (C6), 158.50 (C4), 150.96 (C2), 146.41 (C8), 134.56-128.75 (C^PPh3^), 119.92 (C5), 62.46 (C10), 14.45 (C11). ^31^P NMR, DMF-*d_7_*, δ, ppm: 31.85.





[Au(L_2_)(PPh_3_)] (**2**): Yield: 83%, Anal. Calc. for C_27_H_26_N_4_OPAu (HL_2_ = 6-butyloxy-9*H*-purine): C, 49.86%; H, 4.03%; N, 8.61%. Found: C, 49.43%; H, 4.02%; N, 8.31%. ESI+ MS (methanol, m/z): 215 (calc. 215) [HL_2_+Na^+^], 651 (651) [M+H^+^]. IR (*ν*_ATR_/cm^–1^): 3048m v(C-H)_ar_; 2953m v(C-H)_al_; 2931m; 2869m; 1599s v(C^…^N)_ring_; 1483m; 1465m; 1444m v(C^…^C)_ring_; 1405m; 1380m; 1337m; 1315s v(C_6_-O); 1242m; 1204w; 1174m; 1150m; 1099m v(O-C_10_); 994w; 946m; 869m; 797m; 752w; 660mw; 645m; 584w; 542s v(Au-N); 499s; 443m; 400w; 327m v(Au-P). ^1^H NMR, DMF-*d_7_*, δ, ppm: 8.46, 1H, s, HC^2^, 8.37, 1H, s, HC^8^, 7.72-7.67, 15H, m, HC^PPh3^, 4.57, 2H, m, HC^10^, 1.79, 2H, q, HC^11^, 1.47, 2H, sx, HC^12^, 0.92, 3H, t, HC^13^. ^13^C NMR, DMF-*d_7_*, δ, ppm: 160.11 (C6), 158.98 (C4), 151.33 (C2), 146.13 (C8), 135.20-129.60 (C^PPh3^), 119.94 (C5), 66.37 (C10), 31.19 (C11), 19.32 (C12), 13.94 (C13). ^31^P NMR, DMF-*d_7_*, δ, ppm: 32.30.





[Au(L_3_)(PPh_3_)] (**3**): Yield: 65%, Anal. Calc. for C_26_H_22_N_4_OPAu (HL_3_ = 6-allyloxy-9*H*-purine): C, 49.22%; H, 3.50%; N, 8.83%. Found: C, 48.89%; H, 3.62%; N, 8.35%. ESI+ MS (methanol, m/z): 199 (calc. 199) [HL_3_+Na]^+^, 635 (635) [M+H]^+^. IR (*ν*_ATR_/cm^–1^): 3056m v(C-H)_ar_; 2986m v(C–H)_al_; 2959m; 2806m; 1592s v(C^…^N)_ring_; 1548m; 1477m; 1453m; 1433s v(C^…^C)_ring_; 1386m; 1300m v(C_6_-O); 1193m; 1176m; 1134m; 1099s v(O-C_11_); 1025w; 989m; 929w; 746m; 712m; 690s; 649m; 584m; 543s v(Au-N); 497s; 451m; 433m; 396m; 326m v(Au-P); 253w. ^1^H NMR, DMF-*d_7_*, δ, ppm: 8.41, 1H, s, HC^2^, 8.27, 1H, s, HC^8^, 7.78-7.07, 15H, m, HC^PPh3^, 6.14, 1H, m, HC^11^, 5.45, 1H, d, HC^12a^, 5.20, 1H, d, HC^12b^, 5.12, 2H, d, HC^10^. ^13^C NMR, DMF-*d_7_*, δ, ppm: 159.05 (C6), 158.29 (C4), 152.67 (C2), 148.21 (C8), 135.43-129.49 (C^PPh3^), 134.00 (C11), 118.30 (C5), 117.54 (C12), 66.79 (C10). ^31^P NMR, DMF-*d_7_*, δ, ppm: 32.10.





[Au(L_4_)(PPh_3_)] (**4**): Yield: 72%, Anal. Calc. for C_30_H_24_N_4_OPAu (HL_4_ = 6-benzyloxy-9*H*-purine): C, 52.64%; H, 3.53%; N, 8.19%. Found: C, 52.74%; H, 3.36%; N, 7.95%. ESI+ MS (methanol, m/z): 249 (calc. 249) [HL_4_+Na]^+^, 685 (685) [M+H]^+^. IR (*ν*_ATR_/cm^–1^): 3055m v(C-H)_ar_; 3007w; 2960m v(C–H)_al_; 2850w; 2806m; 2665m; 2569m; 1607s; 1586s v(C^…^N)_ring_; 1492w; 1468m; 1452m v(C^…^C)_ring_; 1399m; 1332m; 1308s v(C_6_-O); 1248m; 1211m; 1175m; 1102m v(O-C_11_); 952m; 915m; 867m; 748m; 725m; 700m; 640m; 578m; 543s v(Au-N); 510m; 496s; 451m; 442m; 433m; 395m; 371m; 327m v(Au-P); 275w. ^1^H NMR, DMF-*d_7_*, δ, ppm: 8.56, 1H, s, HC^2^, 8.50, 1H, s, HC^8^, 7.72, 15H, m, HC^PPh3^, 7.55-7.38, 5H, m, HC^12-16^, 5.46, 2H, s, HC^10^. ^13^C NMR, DMF-*d_7_*, δ, ppm: 159.92 (C6), 159.19 (C4), 152.55 (C2), 148.17 (C8), 136.85 (C11), 135.39-129.30 (C^PPh3^), 128.69-128.13 (C^12-16^), 68.83 (C10). ^31^P NMR, DMF-*d_7_*, δ, ppm: 32.77.





[Au(L_5_)(PPh_3_)] (**5**): Yield: 57%, Anal. Calc. for C_31_H_26_N_4_OPAu (HL_5_ = 6-phenethyloxy-9*H*-purine): C, 53.30%; H, 3.75%; N, 8.02%. Found: C, 52.91%; H, 3.78%; N, 7.71%. ESI+ MS (methanol, m/z): 263 (calc. 263) [HL_5_+Na]^+^, 699 (699) [M+H]^+^. IR (*ν*_ATR_/cm^–1^): 3056m v(C-H)_ar_; 2961m; 2796w v(C-H)_al_; 2581w; 1592s v(C^…^N)_ring_; 1548m; 1451m; 1435s v(C^…^C)_ring_; 1371m; 1335m; 1309s v(C_6_-O); 1260m; 1173m; 1128m; 1099s v(O-C_10_); 997m; 958m; 874w; 798m; 744m; 689s; 645m; 582w; 545s v(Au-N); 500s; 450s; 436m; 329m v(Au-P); 255w. ^1^H NMR, DMF-*d_7_*, δ, ppm: 8.40, 1H, s, HC^2^, 8.23, 1H, s, HC^8^, 7.73-7.69, 15H, m, HC^PPh3^, 7.33-7.18, 5H, m, HC**^13-17^**, 4.77, 2H, t, HC^10^, 3.54, 2H, t, HC^11^. ^13^C NMR, DMF-*d_7_*, δ, ppm: 159.32 (C6), 158.06 (C4), 151.77 (C2), 148.77 (C8), 138.82 (C12), 134.62-129.99 (C^PPh3^), 129.36-126.63 (C^13-17^), 117.57 (C5), 67.03 (C10), 35.02 (C11). ^31^P NMR, DMF-*d_7_*, δ, ppm: 32.61.





[Au(L_6_)(PPh_3_)] (**6**): Yield: 75%, Anal. Calc. for C_25_H_21_N_4_OPClAu (HL_6_ = 2-chloro-6-ethoxy-9*H*-purine): C, 45.71%; H, 3.22%; N, 8.53%. Found: C, 45.97%; H, 3.18%; N, 8.96%. ESI+ MS (methanol, m/z): 221 (calc. 221) [HL_6_+Na]^+^, 657 (657) [M+H]^+^. IR (*ν*_ATR_/cm^–1^): 3053w v(C-H)_ar_; 2980m v(C-H)_al_; 2901w; 1594s v(C^…^N)_ring_; 1534m; 1478w; 1435m; 1420s v(C^…^C)_ring_; 1371m; 1339s v(C_6_-O); 1291m; 1263m; 1205s; 1118s v(O-C_11_); 1101s; 1018m; 995m; 934m; 868m; 748m; 692s; 639m; 636m; 581w; 541s v(Au-N); 513s; 500s; 456m; 449m; 423m; 346m v(Au-P); 256. ^1^H NMR, DMF-*d_7_*, δ, ppm: 8.28, 1H, s, HC^8^, 7.81-7.68, 15H, m, HC^PPh3^, 4.58, 2H, q, HC^10^, 1.36, 3H, t, HC^11^. ^13^C NMR, DMF-*d_7_*, δ, ppm: 160.22 (C6), 159.38 (C4), 151.77 (C2), 150.09 (C8), 134.69-128.60 (C^PPh3^), 120.53 (C5), 63.42 (C10), 14.33 (C11). ^31^P NMR, DMF-*d_7_*, δ, ppm: 31.67.





[Au(L_7_)(PPh_3_)] (**7**): Yield: 52%, Anal. Calc. for C_27_H_25_N_4_OPClAu (HL_7_ = 2-chloro-6-butyloxy-9*H*-purine): C, 47.35%; H, 3.68%; N, 8.18%. Found: C, 47.65%; H, 3.69%; N, 7.84%. ESI+ MS (methanol, m/z): 193 (calc. 193) [HL_7_-Cl+2H]^+^, 651 (651) [M+H]^+^. IR (*ν*_ATR_/cm^–1^): 3052w v(C-H)_ar_; 2958m v(C-H)_al_; 2932m; 2870w; 1601s v(C^…^N)_ring_; 1541m; 1471m; 1451m; 1435m v(C=C)_ar_; 1388m; 1369m; 1333m v(C_6_-O); 1206m; 1183w; 1101s v(O-C_11_); 996w; 960w; 932w; 892w; 801w; 752m; 711w; 692m; 634w; 586w; 543s v(Au-N); 512m; 504s; 443w; 432w; 328m v(Au-P); 225w. ^1^H NMR, DMF-*d_7_*, δ, ppm: 8.42, 1H, s, HC^8^, 7.76-7.67, 15H, m, HC^PPh3^, 4.56, 2H, m, HC^10^, 1.74, 2H, q, HC^11^, 1.44, 2H, sx, HC^12^, 0.84, 3H, t, HC^13^. ^13^C NMR, DMF-*d_7_*, δ, ppm: 160.20 (C6), 159.15 (C4), 151.90 (C2), 150.13 (C8), 135.40-128.71 (C^PPh3^), 119.71 (C5), 66.18 (C10), 31.23 (C11), 19.38 (C12), 13.60 (C13). ^31^P NMR, DMF-*d_7_*, δ, ppm: 32.43.





[Au(L_8_)(PPh_3_)] (**8**): Yield: 46%, Anal. Calc. for C_26_H_21_N_4_OPClAu (HL_8_ = 2-chloro-6-allyloxy-9*H*-purine): C, 46.69%; H, 3.16%; N, 8.38%. Found: C, 46.32%; H, 3.02%; N, 8.68%. ESI+ MS (methanol, m/z): 199 (calc.199) [HL_8_+Na]^+^, 669 (669) [M+H]^+^. IR (*ν*_ATR_/cm^–1^): 3057m v(C-H)_ar_; 2982m v(C–H)_al_; 2960m; 2826m; 1596s v(C^…^N)_ring_; 1528m; 1480m; 1460m; 1432s v(C^…^C)_ring_; 1367m; 1299m v(C_6_-O); 1284m; 1200m; 1135m; 1001s v(O-C_11_); 1030w; 990w; 943m; 750m; 710m; 689s; 589m; 543s v(Au-N); 505s; 449m; 427m; 327m v(Au-P); 250w. ^1^H NMR, DMF-*d_7_*, δ, ppm: 8.43, 1H, s, HC^8^, 7.67-7.23, 15H, m, HC^PPH3^, 6.25, 1H, m, HC^11^, 5.49, 1H, d, HC^12a^, 5.27, 1H, d, HC^12b^, 5.24, 2H, d, HC^10^. ^13^C NMR, DMF-*d_7_*, δ, ppm: 160.54 (C6), 159.47 (C4), 151.31 (C2), 149.15 (C8), 136.28-130.01 (C^PPh3^), 132.80 (C11), 118.50 (C12), 117.29 (C5), 68.19 (C10). ^31^P NMR, DMF-*d_7_*, δ, ppm: 33.13.





[Au(L_9_)(PPh_3_)] (**9**): Yield: 43%, Anal. calc. for C_30_H_23_N_4_OPClAu (HL_9_ = 2-chloro-6-benzyloxy-9*H*-purine): C, 50.12%; H, 3.22%; N, 7.79%. Found: C, 50.43%; H, 3.36%; N, 7.52%. ESI+ MS (methanol, *m/z*): 301 (calc. 301) [HL_9_+K^+^], 719 (719) [M+H^+^]. IR (*ν*_ATR_/cm^–1^): 3095m v(C-H)_ar_; 3051w; 2973w; 2890 v(C–H)_al_; 2679w; 1598 v(C^…^N)_ring_; 1581m; 1549m; 1464m; 1437 v(C^…^C)_ring_; 1355m; 1297s v(C_6_-O); 1231m; 1179m; 1087m v(O-C_11_); 1001w; 957w; 844w; 738m; 693m; 666w; 618w; 581w; 543s v(Au-N); 506s; 458m; 442m; 328m v(Au-P). ^1^H NMR, DMF-*d_7_*, δ, ppm: 8.24, 1H, s, HC^8^, 7.84-7.67, 15H, m, HC^PPh3^, 7.57-7.39, 5H, m, HC^12-16^, 5.50, 2H, s, HC^10^. ^13^C NMR, DMF-*d_7_*, δ, ppm: 160.22 (C6), 159.59 (C4), 151.93 (C2), 148.37 (C8), 136.42 (C11), 135.46-129.98 (C^PPh3^), 128.84-128.37 (C^12-16^), 117.02 (C5), 69.40 (C10). ^31^P NMR, DMF-*d_7_*, δ, ppm: 32.73.





**Table S1.** Crystal data and structure refinements for [Au(L_1_)(PPh_3_)] (**1**) and [Au(L_3_)(PPh_3_)] (**3**).

| Compound | **1** | **3** |
| --- | --- | --- |
| Empirical formula | C_25_H_22_N_4_OPAu | C_26_H_22_N_4_OPAu |
| Formula weight | 622.40  .4,4 | 634.41  .4,4 |
| Temperature (K) | 120(2) K | 120(2) K |
| Wavelength (Å) | 0.71073 Å | 0.71073 Å |
| Crystal system | Triclinic | Triclinic |
| Space group | *P*-1 | *P*-1 |
| *a* (Å) | 10.30067(16) | 10.4415(3) |
| *b* (Å) | 10.84320(18) | 11.08825(19) |
| *c* (Å) | 11.33380(17) | 11.1500(2) |
| *α* (°) | 103.9686(14) | 102.6310(15) |
| *β* (°) | 97.5474(13) | 111.338(2) |
| *γ* (°) | 109.9333(15) | 97.4988(18) |
| *V* (Å^3^) | 1122.75(3) | 1141.56(4) |
| *Z*, *D_calc_* (g cm^–3^) | 2, 1.841 | 2, 1.846 |
| Absorption coefficient (mm^–1^) | 6.648 | 6.541 |
| Crystal size (mm) | 0.40 × 0.30 × 0.25 | 0.40 × 0.30 × 0.30 |
| *F* (000) | 604 | 616 |
| *θ* range for data collection (°) | 2.95 ≤ *θ* ≤ 25.00 | 3.20 ≤ *θ* ≤ 25.00 |
| Index ranges (*h, k, l*) | –12 ≤ *h* ≤ 10 | –12 ≤ *h* ≤ 10 |
|  | –12 ≤ *k* ≤ 12 | –13 ≤ *k* ≤ 13 |
|  | –13 ≤ *l* ≤ 13 | –11 ≤ *l* ≤ 13 |
| Reflections collected/unique | 10429/3948 | 10187/4019 |
| Data/restraints/parameters | 3948/0/290 | 4019/0/301 |
| Goodness–of–fit on *F*^2^ | 1.068 | 1.074 |
| Final *R* indices [*I*>2σ(*I*)] | *R*_1_ = 0.0214, w*R*_2_ = 0.0525 | *R*_1_ = 0.0203, w*R*_2_ = 0.0514 |
| *R* indices (all data) | *R*_1_ = 0.0224, w*R*_2_ = 0.0528 | *R*_1_ = 0.0217, w*R*_2_ = 0.0518 |
| Largest peak and hole (e Å^–3^) | 2.623 and –0.780 | 1.792 and –0.631 |
| CCDC number | 1010556 | 1010557 |

**Table S2.** Selected bond lengths and angles (Å, °) in complexes **1** and **3**.

|  | **1** | **3** |  |  | **1** | **3** |
| --- | --- | --- | --- | --- | --- | --- |
| Au1–N9 | 2.046(3) | 2.042(3) |  | N9–Au1–P1 | 172.73(10) | 175.65(8) |
| Au1–P1 | 2.2344(9) | 2.2356(8) |  | C10–P1–Au1 | 114.58(13) | 114.11(11) |
| P1–C10 | 1.809(4) | 1.811(3) |  | C20–P1–Au1 | 108.70(12) | 109.92(10) |
| P1–C20 | 1.814(4) | 1.816(3) |  | C30–P1–Au1 | 113.98(12) | 113.21(10) |
| P1–C30 | 1.816(4) | 1.820(3) |  | C10–P1–C20 | 106.39(17) | 106.84(14) |
| O1–C6 | 1.346(5) | 1.352(4) |  | C10–P1–C30 | 106.94(17) | 106.57(14) |
| O1–C7 | 1.439(5) | 1.436(4) |  | C20–P1–C30 | 105.62(17) | 105.65(14) |
| N1–C6 | 1.319(5) | 1.323(4) |  | C8–N9–Au1 | 123.8(3) | 125.9(2) |
| N1–C2 | 1.351(5) | 1.354(4) |  | C4–N9–Au1 | 131.5(3) | 130.0(2) |
| N3–C2 | 1.317(5) | 1.321(4) |  | C6–N1–C2 | 117.2(3) | 117.3(3) |
| N3–C4 | 1.350(5) | 1.341(4) |  | C2–N3–C4 | 111.9(3) | 111.6(3) |
| N7–C8 | 1.331(5) | 1.316(4) |  | C8–N7–C5 | 101.0(3) | 102.1(3) |
| N7–C5 | 1.387(5) | 1.381(4) |  | C8–N9–C4 | 104.3(3) | 103.9(3) |
| N9–C8 | 1.327(5) | 1.359(4) |  | C6–O1–C7 | 118.4(3) | 117.0(3) |
| N9–C4 | 1.372(5) | 1.374(4) |  |  |  |  |


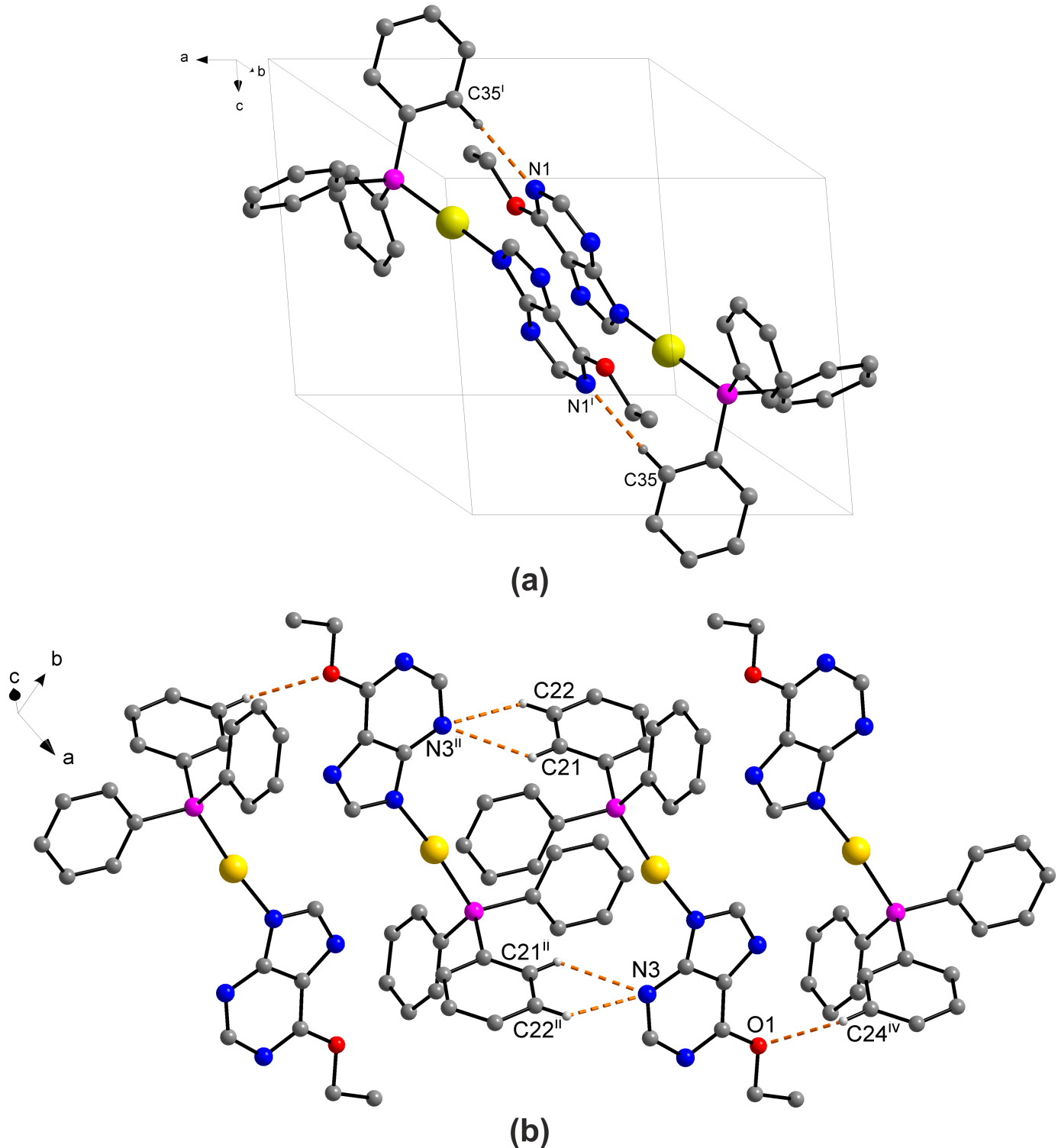


**Figure S1.** Parts of the crystal structure of complex [Au(L_1_)PPh_3_] (**1**) showing **a)** the molecular packing within the unit cell and C–H···N non-covalent contacts (dashed lines); and **b)** the crystal structure stabilization by C–H···O and C–H···N non-covalent contacts (dashed lines).

**Table S3**. Selected non-covalent contacts and their parameters for **1** (Å, °).

| D−H···A | *d*(D−H) | *d*(H···A) | *d*(D···A) | < (DHA) |
| --- | --- | --- | --- | --- |
| C35−H35A···N1^i^ | 0.950 | 2.582(4) | 3.518(6) | 168.7(3) |
| C21−H21A···N3^ii^ | 0.950 | 2.661(3) | 3.230(4) | 125.1(3) |
| C22−H22A···N3^ii^ | 0.950 | 2.711(3) | 3.323(5) | 122.8(3) |
| C23−H23A···N7^iii^ | 0.950 | 2.744(3) | 3.398(6) | 129.7(3) |
| C24−H24A···O1^iv^ | 0.950 | 2.608(2) | 3.306(4) | 130.6(3) |
| C8−H8A···C8^iv^ | 0.950 | 2.717(5) | 3.423(7) | 131.7(3) |
| C34−H34A···O1^v^ | 0.950 | 2.545(4) | 3.463(6) | 162.7(3) |

symmetry codes: (i) 1 – x, 1 – y, 1 – z; (ii) –x, 1 – y, 1 – z; (iii) –1 + x, y, z; (iv) 1 – x, 2 – y, 1 – z;
(v) x, y, 1 + z.


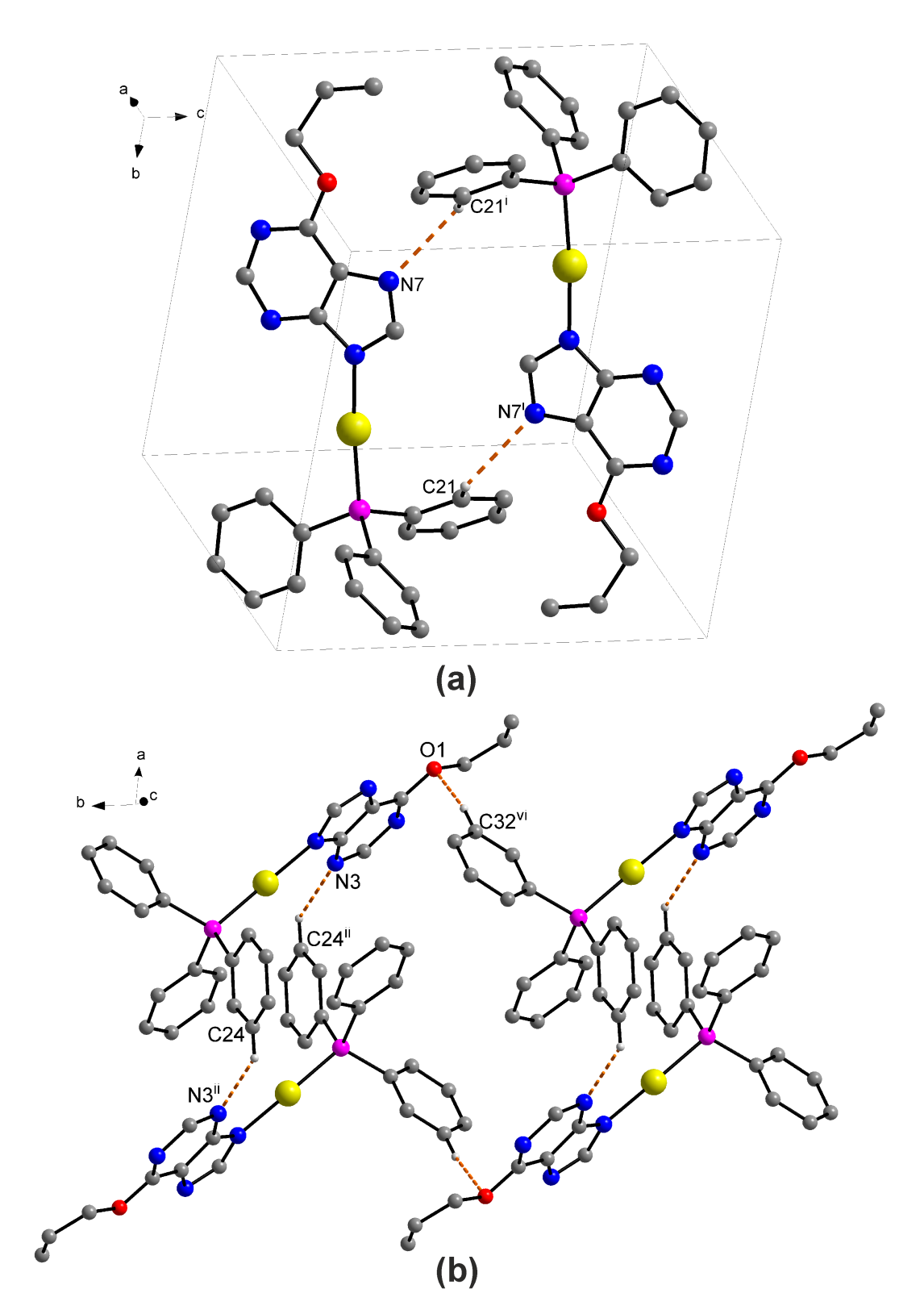


**Figure S2.** Parts of the crystal structure of complex [Au(L_3_)PPh_3_] (**3**) showing **a)** the molecular packing within the unit cell and C–H···N non-covalent contacts (dashed lines); and **b)** the crystal structure stabilization by C–H···O and C–H···N non-covalent contacts (dashed lines).

**Table S4**. Selected non-covalent contacts and their parameters for **3** (Å, °).

| D−H···A | *d*(D−H) | *d*(H···A) | *d*(D···A) | < (DHA) |
| --- | --- | --- | --- | --- |
| C21−H21A···N7^i^ | 0.950 | 2.720(3) | 3.340(4) | 123.6(2) |
| C24−H24A···N3^ii^ | 0.950 | 2.750(3) | 3.381(4) | 124.7(2) |
| C31−H31A···N1^iii^ | 0.950 | 2.581(3) | 3.506(5) | 164.4(2) |
| C32−H32A···O1^iv^ | 0.950 | 2.644(2) | 3.546(6) | 158.6(3) |

symmetry codes: (i) 1 – x, 1 – y, 1 – z; (ii) –x, 1 – y, –z; (iii) 1 – x, 1 – y, –z; (iv) x, 1 + y, z;
(v) 1 – x, –y, –z.


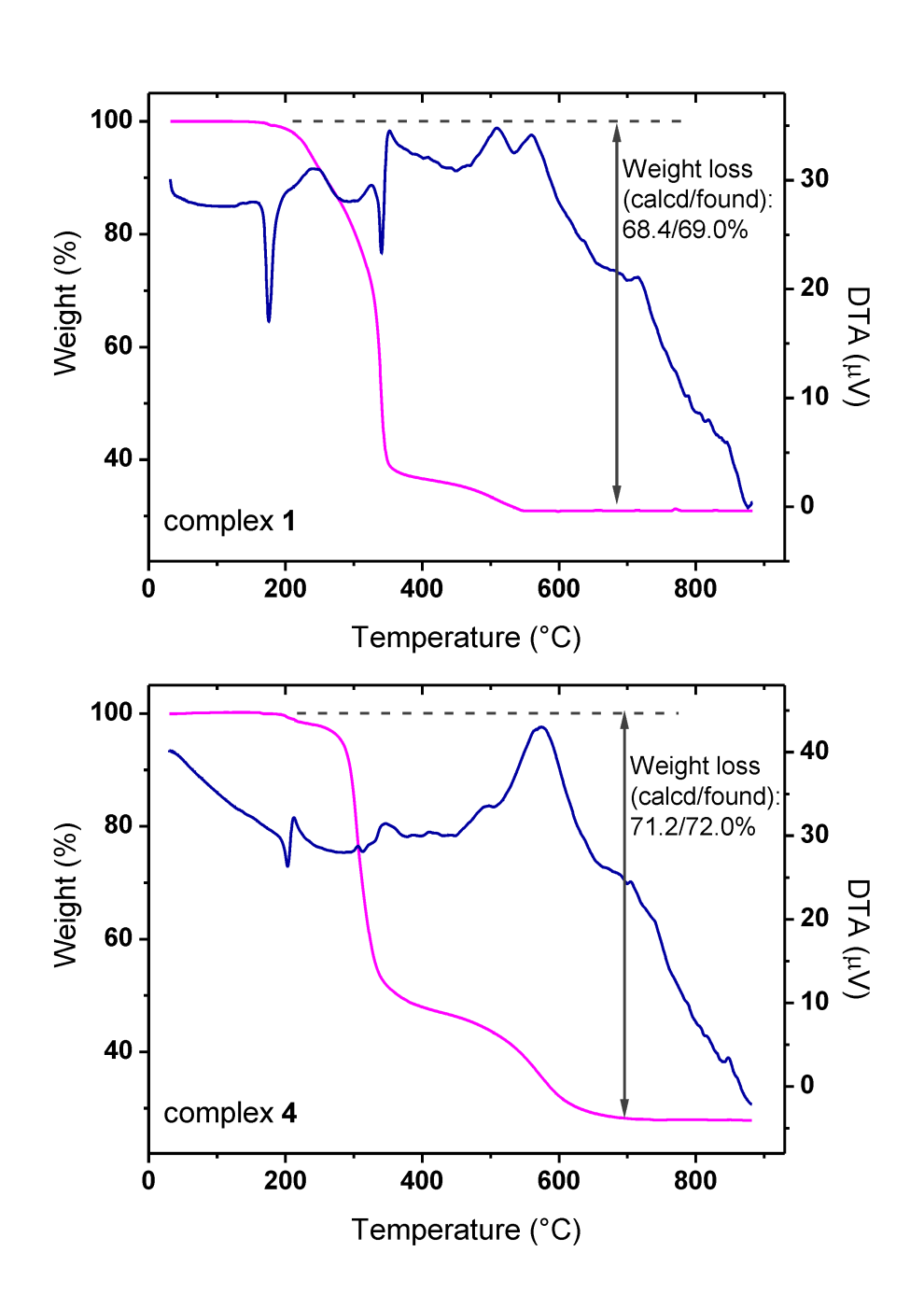


**Figure S3.** TG/DTA curves of the complexes **1** (*up*) and **4** (*down*) given together with the calculated and observed weight losses with Au being the final product of thermal degradation.


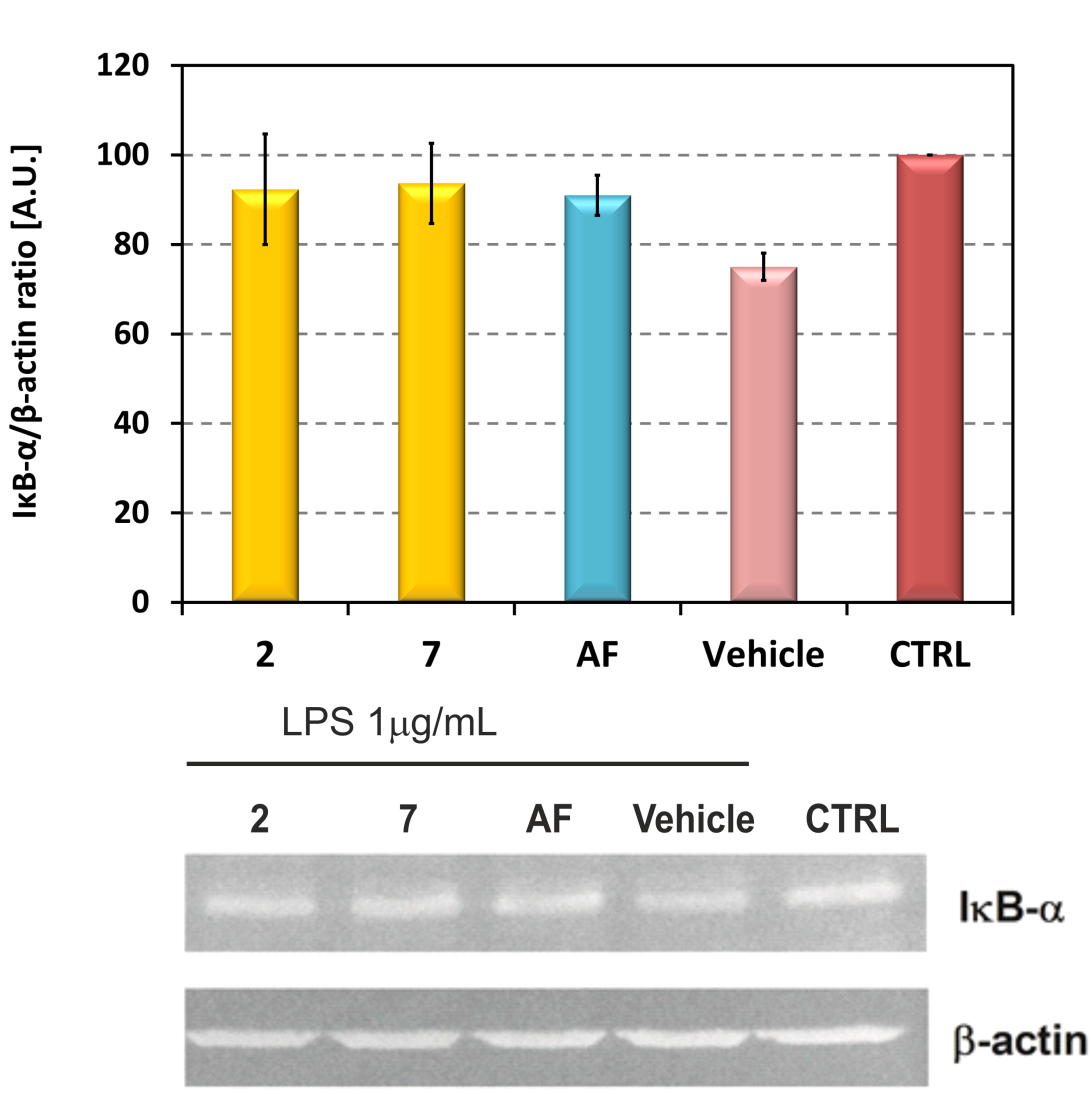


**Figure S4. Effects of the Au(I) complexes, and reference drug Auranofin (AF) on the LPS-induced degradation of I*κ*B-*α*.** The cells were pretreated with the tested compounds (300 nM) or the vehicle (0.1% DMSO) only. After 1h of the incubation, the inflammatory response was induced by LPS [except for the control cells (CTRL)]. The levels of IκB-α and β-actin were determined 30 min after the LPS treatment. The graph (up) shows the IκB-α/β-actin ratio. The results are expressed as means±SE of three independent experiments. The blots (down) show the representative results from three independent experiments.





**Figure S5.** ^31^P NMR spectrum of complex **6**.
